# Ectomycorrhizal and saprotrophic fungi respond differently to long‐term experimentally increased snow depth in the High Arctic

**DOI:** 10.1002/mbo3.375

**Published:** 2016-06-02

**Authors:** Sunil Mundra, Rune Halvorsen, Håvard Kauserud, Mohammad Bahram, Leho Tedersoo, Bo Elberling, Elisabeth J. Cooper, Pernille Bronken Eidesen

**Affiliations:** ^1^ The University Centre in SvalbardP.O. Box 156NO‐9171LongyearbyenNorway; ^2^ Natural History MuseumUniversity of OsloOsloNorway; ^3^ Section for Genetics and Evolutionary BiologyDepartment of BiosciencesUniversity of OsloP.O. Box 1066, BlindernNO‐0316OsloNorway; ^4^ Institute of Ecology and Earth SciencesTartu University14A Ravila50411TartuEstonia; ^5^ Department of Organismal BiologyEvolutionary Biology CentreUppsala UniversitySE 75236UppsalaSweden; ^6^ Natural History MuseumUniversity of Tartu14A Ravila50411TartuEstonia; ^7^ Center for Permafrost (CENPERM)Department of Geosciences and Natural Resource ManagementUniversity of CopenhagenDK‐1350CopenhagenDenmark; ^8^ Department of Arctic and Marine BiologyInstitute of Biosciences Fisheries and EconomicsUiT The Arctic University of NorwayN‐9037TromsøNorway

**Keywords:** Arctic ecology, climate change, fungal richness and communities, Illumina sequencing, Spitsbergen, Svalbard, temporal variation, winter warming

## Abstract

Changing climate is expected to alter precipitation patterns in the Arctic, with consequences for subsurface temperature and moisture conditions, community structure, and nutrient mobilization through microbial belowground processes. Here, we address the effect of increased snow depth on the variation in species richness and community structure of ectomycorrhizal (ECM) and saprotrophic fungi. Soil samples were collected weekly from mid‐July to mid‐September in both control and deep snow plots. Richness of ECM fungi was lower, while saprotrophic fungi was higher in increased snow depth plots relative to controls. [Correction added on 23 September 2016 after first online publication: In the preceding sentence, the richness of ECM and saprotrophic fungi were wrongly interchanged and have been fixed in this current version.] ECM fungal richness was related to soil NO_3_‐N, NH_4_‐N, and K; and saprotrophic fungi to NO_3_‐N and pH. Small but significant changes in the composition of saprotrophic fungi could be attributed to snow treatment and sampling time, but not so for the ECM fungi. Delayed snow melt did not influence the temporal variation in fungal communities between the treatments. Results suggest that some fungal species are favored, while others are disfavored resulting in their local extinction due to long‐term changes in snow amount. Shifts in species composition of fungal functional groups are likely to affect nutrient cycling, ecosystem respiration, and stored permafrost carbon.

## Introduction

The Arctic region has become warmer over the past century (Kaufman et al. 2009). Climate models predict further increase in the rates of warming and precipitation, particularly in the form of snow during winter in polar regions (Moritz et al. 2002; A.C.I.A. 2005; Bintanja and Selten 2014). The amount and density of snow cover can in turn influence Arctic vegetation and soil processes by protecting the ground from abrasive wind and affecting soil insulation during winter (Sturm and Benson 1997; Semenchuk et al. 2013). Such increase in soil temperature will affect the stability of the large carbon (C) pool stored in permafrost soils and nitrogen (N) cycling in moist tundra through elevated microbial metabolic activity (Schimel et al. 2004; Davidson and Janssens 2006; Elberling et al. 2013; Semenchuk et al. 2015). Deeper snow cover may also postpone the onset of spring growth (Cooper 2014). The summed effects of these changes may strongly alter biogeochemical cycling and functioning of the Arctic soil communities (Schimel et al. 2004; Christiansen et al. 2012).

Fungi constitute a major component of the soil microbial community. There are three major fungal functional groups that acquire energy in different ways, namely, parasites, saprotrophs, and mycorrhizal fungi, the two latter of which are especially important for nutrient cycling (Smith and Read 2010). These groups have complementary roles in the cycling of nutrients through soil organic matter (Talbot et al. 2013). Saprotrophic fungi actively decompose organic matter through excretion of a variety of hydrolases, including proteinases, cellulases, and laccases, and thereby increase soil nutrient availability (Baldrian and Valášková 2008). Mycorrhizal fungi assist plants' uptake of nutrients and water from soil and receive C from the host plant in return. Mycorrhizal fungi play a particularly important role in the functioning of Arctic ecosystems, where low water and nutrient availability limit plant growth and productivity (Timling and Taylor 2012). It is estimated that 86% of the N obtained by Arctic plants is via mycorrhizal, notably ectomycorrhizal (ECM), fungi (Hobbie and Hobbie 2006). Nevertheless, our knowledge of the important responses of fungi to ongoing climatic changes in the Arctic is still scarce.

Changes in fungal richness and composition have been found in several summer warming experiments. Clemmensen et al. (2006) and Deslippe et al. (2011) found greater richness and biomass of ECM fungi associated with the arctic‐alpine dwarf shrub *Betula nana*, while Morgado et al. (2014) found lower richness of soil ECM communities in moist tundra. Furthermore, warming altered the composition of the root‐associated ECM fungal community, promoted development of larger mycelia, and triggered changes in nutrient acquisition and belowground carbon transfer (Deslippe and Simard 2011; Deslippe et al. 2011). The contrasting effects of summer warming experiments on ECM fungal richness complicate predicting the effects of winter warming.

Besides warming, increased winter precipitation in the form of snow may cause delayed snow melt and shorter growing seasons. Such changes may affect fungal richness and composition and fruiting patterns (Kauserud et al. 2010). Temporal variation in ECM fungal community structure has been reported in forest ecosystems (Koide et al. 2007), possibly as a result of delayed growth initiation due to water stress (Coleman et al. 1989), responses to temperature (Tibbett et al. 1998), and nutrient availability (Lilleskov et al. 2002). Recently, Mundra et al. (2015a) also found high spatial heterogeneity in the Arctic fungal communities, both in winter and summer period, possibly due to variation in local microclimatic and topographic conditions, which is a common phenomenon in the Arctic (Washburn 1980). They also reported that spatial heterogeneity affected temporal pattern of fungal richness and communities. Filling the gaps in our knowledge of the temporal dynamics of Arctic ECM fungal communities is important to be able to predict the effects of expected climate change. In particular, it is important to know how delayed snow melt, induced by increased snow depth, affects fine‐scale temporal variation in fungal richness and community structure.

In this study, our main aim is to examine (1) how snow depth influences fungal richness and composition in Arctic soils and (2) how snow depth, associated soil and microclimate factors affect the temporal variation in fungi during the growing season. We also explored the response of different functional groups of fungi (ECM and saprotrophic) to alter snow depth. We utilized a snow accumulation experiment established by Cooper and colleagues (Morgner et al. 2010; Cooper et al. 2011) in 2006 in Adventdalen, Svalbard, in which snow fences are used to manipulate snow accumulation and snow distribution patterns.

## Materials and Methods

### Site description and experimental setup

The study was carried out in Adventdalen, a wide river valley on Spitsbergen, the largest island in the High Arctic archipelago Svalbard, Norway (78°10′N, 16°06′E, 40 m asl). The vegetation at the study site was a mesic meadow, dominated by the evergreen dwarf shrubs *Dryas octopetala* and *Cassiope tetragona*, the deciduous dwarf shrub *Salix polaris*, graminoids (mainly *Luzula confusa*,* Alopecurus magellanicus*, and *Carex* spp.), herbs, and mosses. The average annual temperature and total precipitation at Svalbard airport (*ca*. 15 km from the site) were −4.6°C and 199 mm, respectively, for the period of 1981–2010 (www.eklima.no). For more details about vegetation and environmental conditions at the site, see Cooper et al. (2011) and Morgner et al. (2010).

Cooper et al. (2011) established 12 split plots of paired fence and control treatments in two vegetation types (heath and meadow habitat), covering 2.5 km × 1.5 km area (see aerial arrangements of fences in Fig. S5). The effects of increased winter snow depth and associated delay of snow melt on fungi were tested by use of an experimental setup with six snow fences (C7, C8, C9, D10, D11, and D12; 6‐m long and 1.5‐m high; located in meadow habitat) each paired with a control area so that the total number of sampling areas was 12 ([fence area + control area] × 6). The location of individual fence (deep snow) and paired control is referred as “FPC location” throughout the article (i.e., six FPC locations). Snow fences reduce wind speed and cause deposition of wind‐transported snow on the lee side. Maximum snow depths of 1.5 m were observed *ca*. 3–12 m behind fences, while the snow depth in control plots was 10–35 cm (Morgner et al. 2010; Cooper et al. 2011). Winter soil temperatures under deep snow areas were overall higher and more stable than in control plots (Semenchuk et al. 2013). In 2012, snow melted (date at which 50% fence area was snow free) on 5th June in the control plots and 19th June in the deep snow plots behind the fences (see Semenchuk et al. 2013, for details about snow melt out date calculations).

### Sample collection and environmental data recording

In each of the 12 sampling areas, a small plot (0.5 × 0.5 m) was established for collection of soil throughout the experiment. Deep snow treatment plots were placed at maximum snow depth behind each fence. One soil sample was collected from each plot weekly from peak growing season until well past plant senescence, in total nine times (20 July, 31 July, 8 August, 16 August, 22 August, 31 August, 7 September, 15 September, and 20 September; year 2012). In total, 108 soil samples were collected. The first sample was taken 45 days and 31 days after snow melt in control and deep snow areas, respectively.

Soil samples were taken by coring, using a steel cylinder 5 cm in diameter. From each core, aboveground plant material (living and litter) was first removed while keeping the structure of the core intact. Thereafter, the soil was divided horizontally into 0–2 and 2–5 cm depth sections, each of which was split vertically into two parts, one for soil chemical and physical analysis and the other for DNA extraction. The latter was stored at −20°C until DNA extraction. In this article, we used the soil chemistry (pH, NO_3_‐N, NH_4_‐N, total organic nitrogen “TON,” total organic carbon “TOC,” and potassium “K”) and water content data for each core section, published in Semenchuk et al. (2015).

Samples from the two depth sections were combined before the final analyses. This was motivated by (1) studies of forest systems failing to detect depth‐specific variation in fungal community structure in the 0–20 cm depth interval (Tedersoo et al. 2006; Talbot et al. 2014); (2) analyses of soil chemistry by Semenchuk et al. (2015) revealing no differences between the 0–2 and 2–5 cm depth intervals; and (3) a global nonmetric multidimensional scaling (GNMDS) ordination analysis of fungal species composition in a dataset consisting of all samples from the 0–2 cm, and a subset of samples from 2 to 5 cm depth (alternate sampling dates), in which no effect of sampling depth was detected (Table S7).

Daily weather data (precipitation, air temperature, and cloud cover) for the meteorological station at Svalbard airport were obtained from www.eklima.no. Averages of air temperature, cloud cover, and cumulative precipitation over the 7‐day period prior to each sampling date were used in the analyses (Table S1).

### DNA extraction and Illumina sequencing

Total soil genomic DNA was extracted from soil using the PowerSoil^®^ DNA Isolation Kit (MO BIO Laboratories, Carlsbad, CA), according to the manufacturer's protocol. One negative extraction control was run in parallel with DNA extraction from samples, and used in sequencing. At the final step, DNA was eluted in 60 *μ*L of elution buffer. For the 25 *μ*L PCR reaction, 4 *μ*L of DNA extract, 11.93 *μ*L of MQ water, 2.50 *μ*L of 10x Dreamtaq buffer, 2.50 *μ*L dNTP's (2.5 mmol/L of each nucleotide), 1.25 *μ*L of reverse and forward primers (10 *μ*mol/L), 1.25 *μ*L BSA (10 *μ*g/*μ*L), and 0.33 *μ*L Dreamtaq polymerase (5U/*μ*L) were used. Modified forward primers fITS7a (Ihrmark et al. 2012) (“A” is inserted instead of “R” at position 5) and reverse primer ITS4 (White et al. 1990) were used to amplify the ITS2 region of the nuclear ribosomal rDNA repeat, using the following PCR conditions: 95°C for 2 min, then 24 cycles of 95°C for 30 sec, 55°C for 30 sec, and 72°C for 1 min, ending with 72°C for 30 min. To increase the multiplexing of the samples, a two base pair (bp) tag was attached at the 5′ end of both forward and reverse primers. Illumina sequencing introduces sequence‐specific error due to phasing caused by specific sequence patterns (Nakamura et al. 2011). Therefore, sample‐specific multiplex identification DNA tags (MIDs) of varying size (4–8) were designed to correct this bias to some extent. These MIDs were attached at both ends of the Illumina‐specific adapter. One end (5′) of the adapter was phosphorylated and at the other end (3′) a “T” base was overhanged, to anneal with “A” overhanged on the PCR‐amplified product. Solid‐phase reversible immobilization (SPRI) beads were used for the purification of amplified PCR products. Paired‐end (PE) adapters oligo mix were ligated to the fragments using T4 DNA ligase (New England Biolabs Ipswich, Massachusetts, USA). To cut “ideoxyU,” located at the center of the Illumina adapter, 1 *μ*L USER enzyme (New England BioLabs) was used. QPCR was used for library quantification to find the optimal end point (low C_*T*_ value) of the PCR cycle, followed by 5′‐end adaptor extension and library‐enrichment PCR with 11 cycles, using Q5^®^ high‐fidelity DNA polymerase enzyme. PE sequencing (2 × 300) was performed in two sequencing plates on an Illumina Miseq sequencing machine. Sequence data, mapping files, and associated metadata are available in Dryad public repository (doi: 10.5061/dryad.r7pc5).

### Bioinformatics analyses

Of the 21,919,080 PE forward and reverse reads, 10,140,279 were assembled using fastq‐join (Aronesty 2013), with a minimum 125‐bp overlap. Initial quality filtering of the joined reads was carried out using the online platform Galaxy (https://usegalaxy.org/). These were quality filtered using filter fastq to discard reads having nucleotide base with quality scores (Q) <20, then filtered by quality (>90% of nucleotide bases with Q36) before removal of sequencing artifacts by the function implemented in the Galaxy, as part of the FASTX‐Toolkit (A. Gordon, http://hannonlab.cshl.edu/fastx_toolkit/). A total of 4,859,204 reads were retained. Reads with length <200 bp and >550 bp, homopolymers exceeding 8 bp, ambiguous base call >0, and >1 mismatch in the forward primer sequence were removed from the dataset, using the split_library.py function implemented in QIIME v. 1.8.0 (Caporaso et al. 2010). In addition, a 50‐bp sliding window was used to identify regions of low sequence quality (average Q <35) and truncate affected sequences at the beginning of the low‐quality window. Illumina sequencing PE reads are randomly attached to the sequencing lane; hence, output fastq files contain ~50% reads in each direction. Therefore, in order to reorient all reads in the same direction, 3′–5′ reads were reverse complemented after demultiplexing. In total, 4,210,067 quality‐filtered reads were exercised for de novo chimera checking using the usearch61 algorithm, with the minimum divergence parameter = 1 and abundance skew = 2 (Edgar 2010). Thereafter, 4,124,789 reads were retained. Nonchimeric reads were clustered into 4956 operational taxonomic units (OTUs) at the 97% similarity threshold using the uclust algorithm and the most abundant sequence of each cluster was designated as the representative sequence (Edgar 2010). Clusters represented by <5 sequences (2122 OTUs; 3713 reads) were discarded to avoid the inclusion of sequencing errors (Nguyen et al. 2014). The representative sequence of each cluster was subjected to BLASTn search against the quality‐checked UNITE+INSD fungal ITS sequence database (release 2 February 2014), containing both identified and unidentified sequences (Kõljalg et al. 2013). OTUs (1) that did not have similarity to fungal sequences in the UNITE database (508 OTUs; 55821 reads); (2) were detected in extraction control (2 OTUs; 4368 reads); (3) <100 bp length (37 OTUs; 1103 reads); and (4) bit‐score/sequence length <0.6 (234 OTUs; 14997 reads) or strictly associated with only one of the samples (16 OTUs; 9207 reads) were excluded from further analysis. The resulting OTU table was rarefied to an even sampling depth of 3069 reads per sample. Each OTU was further classified into their functional category by referring Tedersoo et al. (2014) supplementary dataset, Tedersoo and Smith (2013), and other available literature. At this stage, the whole dataset (“Total fungi”) was divided into two subsets based on functional groups: the “ECM fungi” and “saprotrophic fungi” datasets. The three datasets (total, ECM, and saprotrophic fungi) were analyzed separately. Considering the potential bias involved in using high‐throughput sequencing quantitative data (Amend et al. 2010; Baldrian et al. 2013), present–absent OTU matrices were used in further downstream analyses. Rarefaction curves for ECM and saprotrophic fungi were calculated using the approach of Ugland et al. (2003).

### Statistical analyses

All statistical analyses were performed in R, version 3.1.2 (R Core Development Team 2014). Soil variables were transformed to zero skewness following Økland et al. (2001). Differences in soil properties, fungal taxonomic OTU richness between treatments, and OTU richness between treatments for each sampling date were tested by use of Student's *t* test, with Benjamini–Hochberg FDR correction of *P* values (Benjamini and Hochberg 1995). Generalized linear mixed models (GLMM) and fit by maximum likelihood with Poisson distribution (Breslow and Clayton 1993) were used to evaluate the direct effects of treatment, sampling date, and their interaction (all treated as fixed effects) on fungal OTU richness, using the *lme4* package (Bates et al. 2014). FPC locations were used as random variable in these GLMM analyses. While analyzing the treatment effect, sampling date was used as an additional random variable. The effect of environmental variables was evaluated by adding them to models by a forward selection procedure by which the *F*‐ratio test was used to compare nested models. At each step in the model selection procedure, *P* values were corrected for multiple testing by the Bonferroni correction (Legendre and Legendre 2012). Finally, treatment was added to the forward selected model and, if significant, interaction terms between the significant environmental variables and treatment were tested for significance. GLMM was also used to test effects of treatment, time, and their interaction (fixed effects) on the taxonomic richness of ECM and saprotrophic fungi (fence location was then used as random variable).

If not otherwise is stated, community analyses were performed on each of the 3 OTU matrices (total, ECM, and saprotrophic fungi) using Bray–Curtis distance measurement. The gradient of community structure was summarized by parallel application of GNMDS (Kruskal 1964; Kruskal et al. 1973) and detrended correspondence analysis (DCA; Hill and Gauch 1980) ordinations using the *vegan* package (Oksanen et al. 2013; see Mundra et al. 2015b, for details of options and settings applied). All ordinations were inspected for known artifacts such as arch effects (in GNMDS), tongue effects (in DCA), and strong outliers (Økland 1990). A reliable gradient structure was inferred if similar results were obtained by the two methods, and no obvious artifacts were seen (Økland 1996; van Son and Halvorsen 2014). Similarity of ordinations was evaluated by calculating Kendall's rank correlation coefficient (*τ*) between DCA and GNMDS axes. Axes were considered acceptably similar if |*τ*| > 0.4 (Liu et al. 2008). The GNMDS ordinations were chosen for further analysis due to lack of artifacts. Interpretation was performed by calculation of *τ* between GNMDS axes and each explanatory variable and by using the *envfit* function in *vegan*, by which each explanatory variable is separately regressed on GNMDS axes 1 and 2 by linear regression analysis. The strength of the relationship between each variable and the ordination axes was assessed by the multiple coefficient of determination (*R* ^2^); Bonferroni correction of *P* values was applied. Effects of fence treatment and sampling date and their interaction on community structure were tested by using multivariate permutational analysis of variance (PERMANOVA) as implemented in the Adonis function of the package *vegan*.

To partition the variation in community dissimilarity variation, explained by deep snow treatment, sampling date, and environmental variables (including soil and weather variables), we used the *varpart* function (based on redundancy analysis, RDA) in the *vegan* package with 999 permutations cycles. To measure the nestedness of species‐poor assemblages (occurring at specific dates) in otherwise species‐rich communities, we used the NODF index, calculated using function *nestednodf* with order = TRUE (values of 0 = non‐nestedness, 100 = perfect nesting) as implemented in *vegan*. We calculated NODF with 1000 permuted matrices constructed from a null model of fixed rows and columns.

## Results

### Soil and weather data

Soil NH_4_‐N, NO_3_‐N, and K concentrations were significantly higher in plots of greater snow depth than in controls plots (all t > −3.90, *P* < 0.001, *n* = 155, unpaired; Fig. S1). Weather conditions such as air temperature, cloud cover, and cumulative precipitation varied temporally throughout the study period, but a combination of moist and warm weather was registered throughout the week preceding 8 August and 16 August (Table S1). Soil water and NO_3_‐N content varied significantly between FPC locations, being relatively lower in D11 and D12 (Table S2).

### Sequence data characteristics

The total fungi dataset (including ECM, saprotrophs, as well as unclassified OTUs) comprised 1760 OTUs (475,695 reads) after read normalization, of which the ECM subset comprised 648 OTUs (37% of total; 340,035 reads) and the saprotrophic subset 343 OTUs (19% of total; 38,595 reads; Table 1). The average number of fungal OTUs recovered per sample was 139.4 ± 30.4 (mean ± SD), 53.6 ± 18.2 for ECM fungi and 28.2 ± 9.6 for saprotrophic fungi. Species accumulation curves for ECM fungi did not approach an asymptote, while the curve for saprotrophic fungi showed a tendency to level off (Fig. S2).

**Table 1:** Sequencing reads and the number of the operational taxonomic units (OTUs) associated with total, ectomycorrhizal (ECM), and saprotrophic fungal dataset

	Total	ECM	Saprotrophic
Total reads	475,695	340,035	38,595
% Reads for top 20 OTUs	57.9	78.6	70.7
% Reads for top 100 OTUs	87.5	98.4	96.2
Total OTUs	1760	648	343
% Singleton OTUs	21.3	21.9	20.7
% Doubleton OTUs	14.5	13.7	13.7

In the total fungi dataset, 48% of the OTUs were assigned to Basidiomycota and 28% to Ascomycota. Zygomycota (1%), Chytridiomycota (1%), and Glomeromycota (<1%) were also detected, whereas 22% were unclassified to phyla. The most OTU‐rich orders were Agaricales (23%), Thelephorales (13%), and Helotiales (6%). The ECM dataset was dominated by Basidiomycota, to which 97% of all OTUs in this dataset were assigned. In total, 17 ECM genera were detected, among which the dominating were *Tomentella* (51%), *Cortinarius* (30%), and *Inocybe* (17%). The saprotrophic dataset was dominated by ascomycetes (47%) and basidiomycetes (40%), while 13% of OTUs were assigned to Zygomycota. OTUs belonging to 40 different orders were detected, among which the dominating were: Agaricales (28%), Mortierellales (13%), and Chaetothyriales (12%). A much higher number of genera were detected in the saprotrophic than in the ECM dataset (107 vs. 17; Table S3).

### Treatment effects on richness and taxonomic composition

**Figure 1:**
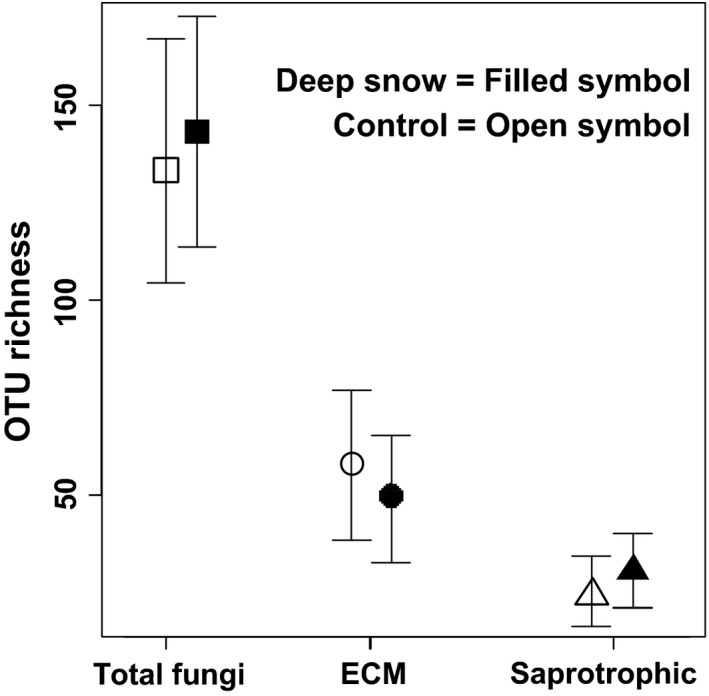
Snow treatments (deep snow vs. control) effect on total, ectomycorrhizal (ECM), and saprotrophic fungal operational taxonomic unit richness (mean ± SD). Generalized linear mixed models showed significant effects of deep snow treatment on total (*z* = 4.4, *P* < 0.001), saprotrophic (*z* = 6.1, *P* < 0.001), and ECM fungi (*z* = −7.6, *P* < 0.001).

The GLMM analyses revealed significant snow treatment effects on fungal richness. The total fungal richness increased with increased snow depths (*z* = 4.4, *P* < 0.001). A similar trend was observed for saprotrophic fungi (*z* = 6.1, *P* < 0.001), while the number of ECM OTUs decreased (*z* = −7.6, *P* < 0.001) when snow depth increased (Fig. 1). The GLMM analysis revealed that the total fungal richness, as well as the richness of saprotrophic fungi alone, was significantly and positively related to soil pH (Table 2). The ECM fungal richness was significantly influenced by soil TON, K, NO_3_‐N, and treatment:NO_3_‐N interaction. A significant treatment effect persisted also after the variation attributed to soil variables had been accounted for (Table 2).

**Table 2:** Fixed effects table for the generalized linear mixed model (GLMM) fitted to the number of operational taxonomic units (OTUs) detected in the samples for total, ectomycorrhizal (ECM), and saprotrophic fungi

	Estimate	SE	*z*	*P*
Total fungi				
Intercept	4.8164	0.0360	133.8	<0.001
Soil pH	0.1613	0.0358	4.5	<0.001
Soil NO_3_‐N	0.0415	0.0383	1.1	0.278
Deep snow	0.0542	0.0161	3.4	<0.001
ECM fungi				
Intercept	3.7901	0.0782	48.5	<0.001
Soil TON	0.5575	0.0695	8.0	<0.001
Soil K	−0.3039	0.0744	−4.1	<0.001
Soil NO_3_‐N	0.2387	0.0796	3.0	0.003
Treatment:NO_3_‐N	0.1509	0.0648	2.3	0.020
Deep snow	−0.6460	0.1212	−5.3	<0.001
Saprotrophic fungi				
Intercept	3.0906	0.0550	56.2	<0.001
Soil NO_3_‐N	0.1070	0.0872	1.2	0.220
Soil pH	0.2401	0.0780	3.1	0.002
Deep snow	0.1701	0.0363	4.7	<0.001

GLMM models were run using soil variable and treatment (deep snow vs. control) as fixed‐effect predictor, while location of fence and paired control (FPC locations) were included as a random factor in the model.

Deep snow treatment significantly reduced the number of ECM OTUs belonging to Basidiomycota, at all taxonomic levels, from orders (Sebacinales and Thelephorales) to genera (*Sebacina* and *Hebeloma*; Table 3). Dominant ECM genera such as *Tomentella*,* Cortinarius*, and *Inocybe* were, however, not affected by snow depth treatment. In contrast, deep snow treatment significantly enhanced the OTU richness of saprotrophic basidiomycetes, while ascomycetes and zygomycetes were not affected at phylum level. Positive treatment effects were evident for both basidiomycetes and ascomycetes at the class level (Agaricomycetes and Dothideomycetes), orders (Agaricales, Dothideales, and Auriculariales), families (Tricholomataceae, Clavariaceae, and Dothideaceae), and genera (*Ramariopsis* and *Dothidea*; Table 3). For saprotrophic fungi, rare OTUs belonging to genera *Kurtzmanomyces*,* Clavulinopsis*,* Sydowia*,* Crocicreas*,* Clathrosporium*,* Lemonniera*, and *Hyphoderma* were only found in control samples (Table S3).

**Table 3:** Average operational taxonomic unit (OTU) richness of different taxonomic level per treatment (deep snow vs. control) for ectomycorrhizal (ECM) and saprotrophic fungi

Taxonomic group	Deep snow	Control	CI (lower)	CI (upper)	*t*	*P*
ECM fungi						
Basidiomycota	47.48	55.97	2.84	14.15	2.97	0.007
Agaricomycetes	47.48	55.97	2.84	14.15	2.97	0.014
Sebacinales	0.77	1.29	0.29	0.77	4.34	<0.001
Thelephorales	15.38	18.31	0.79	5.07	2.71	0.026
Sebacinaceae	0.77	1.29	0.29	0.77	4.34	<0.001
Strophariaceae	1.49	1.99	0.16	0.83	2.91	0.018
Thelephoraceae	15.38	18.31	0.79	5.07	2.71	0.022
Cortinariaceae	13.86	18.42	0.72	8.41	2.34	0.046
Sebacina	0.77	1.29	0.29	0.77	4.34	<0.001
Hebeloma	1.49	1.99	0.16	0.83	2.91	0.025
Saprotrophic fungi						
Basidiomycota	12.73	9.78	−4.31	−1.58	−4.267	<0.001
Agaricomycetes	10.66	8.18	−3.67	−1.30	−4.145	<0.001
Dothideomycetes	3.78	2.83	−1.71	−0.18	−2.437	0.037
Agaricales	8.83	6.94	−2.91	−0.88	−3.678	0.005
Dothideales	1.53	0.87	−1.07	−0.25	−3.152	0.015
Auriculariales	0.65	0.37	−0.48	−0.07	−2.67	0.042
Tricholomataceae	3.42	2.51	−1.41	−0.39	−3.495	0.013
Clavariaceae	2.06	1.23	−1.33	−0.34	−3.357	0.011
Dothideaceae	1.49	0.83	−1.06	−0.26	−3.26	0.010
Ramariopsis	1.49	0.76	−1.09	−0.38	−4.11	0.001
Dothidea	1.47	0.83	−1.03	−0.23	−3.13	0.023

Significance of snow treatment effect was determined using the Student's *t* test at the 5% level, followed by FDR correction of *P* values. Only significantly varying taxonomical groups with mean OTU richness and 95% confidence intervals (CI) are shown. Each taxonomic groups (phylum, class, order, family, and genera) with >1% of total OTU frequency (Table S8) within each treatment category are considered for analysis.

### Richness variation between treatments with time

The GLMM analyses revealed that the total fungal richness, as well as the richness of saprotrophic and ECM fungi alone, varied temporally. The total fungal richness, as well as the richness of saprotrophic fungi peaked on 16th August (Fig. S3), after a period with warmer and moister weather conditions (Table S1).

**Figure 2:**
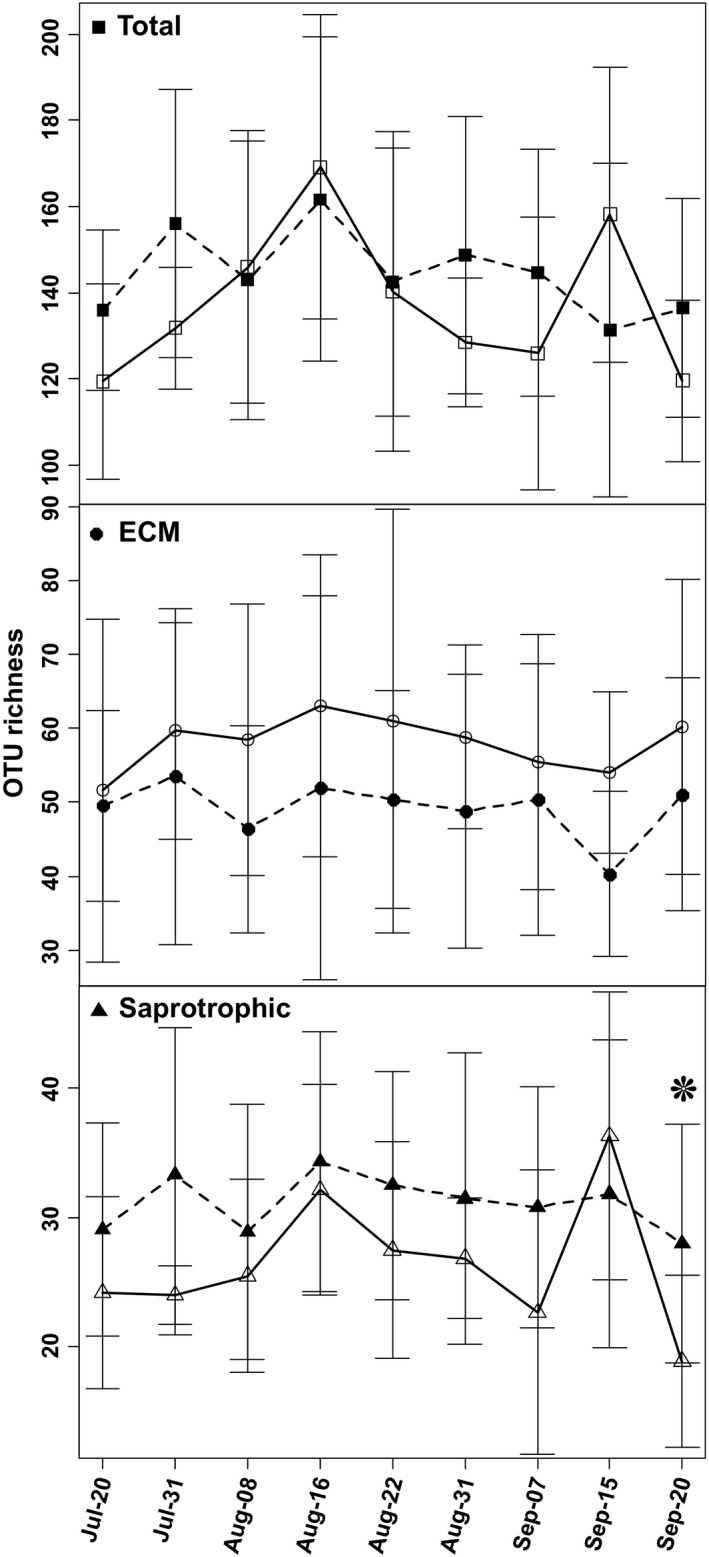
Snow treatments (deep snow vs. control) effect on pattern of temporal variation in operational taxonomic unit richness (mean ± SD) for total, ectomycorrhizal, and saprotrophic fungi analyzed using generalized linear mixed models. Open symbol and solid line indicate control and filled symbol and dashed line indicate deep snow. Star symbol indicates significant variation (determined by *t* test) between treatments for particular sampling date.

The GLMM analysis demonstrated different patterns of temporal variation in OTU richness between deep snow and control plots for all three datasets (Table S4, Fig. 2). The total fungal richness was relatively higher (although nonsignificant) in deep snow plots than in control plots at the start of the study (20 July). Saprotrophic fungal richness mostly followed the pattern observed for total fungal richness, but on 20th September there was a significant difference between deep snow treatment and control (*t* = −2.6, *P* = 0.01, *n* = 21, unpaired). For saprotrophic fungi, particularly strong differences in temporal variation between treatment and control were observed for the taxonomic groups Ascomycetes, Leotiomycetes, and Helotiales (Table S5). For ECM fungi, responses differed little among taxonomic groups.

### Community structure: treatments effects and temporal variation

**Figure 3:**
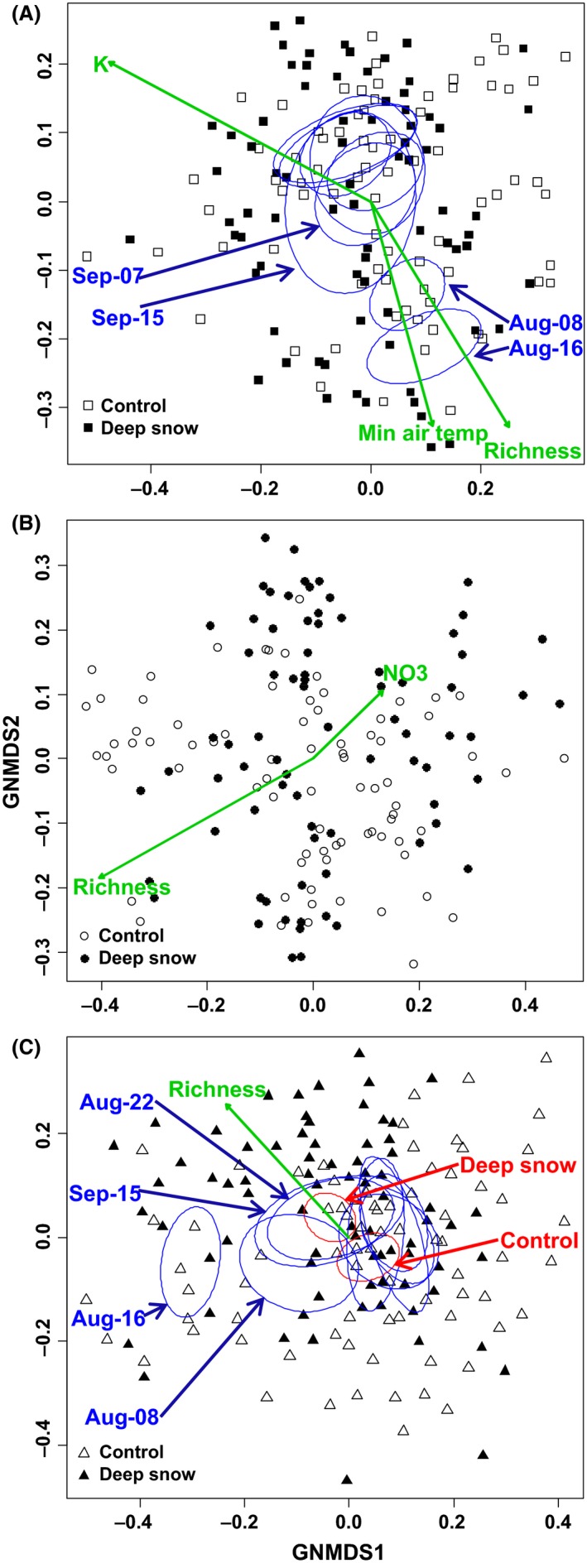
Global nonmetric multidimensional scaling ordinations of operational taxonomic units included in the three datasets (A) total, (B) ectomycorrhizal, and (C) saprotrophic fungal composition, where (B) and (C) represent subsets of (A). Soil samples were collected weekly from mid‐July to mid‐September from deep snow and control snow plots. The arrows point in the direction of maximum increase for each explanatory variable, which are statistically significant after Bonferroni correction. Ellipses indicate 95% confidence intervals around centroids of each category and are shown if they explain significant variations. Ellipses with red color indicate treatment effect and blue color represent sampling date effect. Axes are scaled in half‐change (H.C.) units.

For each of the three fungal datasets, both axes of the two‐dimensional GNMDS ordinations were strongly correlated with the corresponding DCA axes (|*τ*| > 0.5, *P* < 0.001; Table S6). They were therefore accepted as representing the main compositional gradients in the datasets. Only a weak treatment effect on saprotrophic fungal OTU composition was found in the GNMDS ordination, while no effect was detected in the analyses of the total and ECM fungi datasets (Table S7). The dataset including all fungi was weakly structured according to sampling date (*R* ^2^ = 0.21; *P* = 0.001) and soil K (*R* ^2^ = 0.12; *P* = 0.001; Fig. 3A). Minimum air temperature covaried with sampling date and was weakly related with all fungal community dataset. Likewise, saprotrophic fungal OTU composition was structured according to sampling date (*R* ^2^ = 0.20; *P* = 0.001; Fig. 3C). In contrast, ECM fungal communities were not structured by sampling date (Fig. 3B, Table S7) but rather by FPC locations (*R* ^2^ = 0.33; *P* = 0.001) and NO_3_‐N content (*R* ^2^ = 0.07; *P* = 0.006).

The Adonis analyses indicated a consistent community structural response to deep snow treatment and significant compositional differences among time points (not significant for ECM fungi), while the interactions between treatment and time points were nonsignificant (Table 4). The variation partitioning analyses further demonstrated that ECM communities were not affected by sampling date, but were weakly influenced by environmental variables (Fig. S4).

**Table 4:** Relative importance in terms of the amount of variance explained by the variables of snow treatment (deep snow vs. control), time (sampling date), and their interaction effect on the total, ectomycorrhizal (ECM), and saprotrophic fungal community structure as revealed from the Adonis analysis

Fungal group	Variable	F.Model	*R* ^2^	Pr(>F)
Total fungi	Treatment	2.930	0.019	**0.001**
	Time	1.536	0.078	**0.001**
	Treatment:Time	0.754	0.038	1
	Residuals	0.866		
ECM fungi	Treatment	2.261	0.015	**0.004**
	Time	0.753	0.040	0.994
	Treatment:Time	0.682	0.036	1
	Residuals	0.909		
Saprotrophic fungi	Treatment	3.049	0.019	**0.001**
	Round	1.644	0.082	**0.001**
	Treatment:Time	0.810	0.041	0.962
	Residuals	0.858		

Statistically significant values are indicated in bold text.

Nestedness analyses demonstrated that communities from sampling dates with low species richness were moderately nested within sampling dates with high species richness (NODF = 60.52 [total fungi], 61.72 [ECM], and 59.92 [saprotrophic]).

## Discussion

Our results suggest that alteration of snow regimes over several years influences soil fungal communities in Arctic mesic meadows, however, with contrasting effect between various fungal groups. The richness of saprotrophic fungi responded positively, while the richness of ECM fungi responded negatively to increased snow depth relative to controls. Increasing richness of saprotrophic fungal species may promote the rate of decomposition of soil organic matter (Setälä and McLean 2004; van der Wal et al. 2013), which in turn affects nutrient cycling (Schimel et al. 2004; Deslippe and Simard 2011).

Despite the significant variation in soil conditions between deep snow and control plots, the temporal variation in soil fungal communities were weakly affected by the treatments, and the temporal effect differed between ECM and saprotrophic fungi. This suggests that increase of snow cover and delay of snow melt may have little impact on the temporal variation in fungal communities.

Saprotrophic fungal OTUs comprised a smaller fraction of the total fungi dataset than ECM (Table 1). Nevertheless, the main pattern of variation in the total fungi dataset closely resembles the pattern seen for saprotrophic fungi, both regarding the effect of snow treatment and sampling date on fungal richness and composition. This possibly suggests that a larger fraction of the unclassified fungi included in the total fungi dataset are saprotrophic fungi, which is not unexpected given the large number of microscopic saprotrophic fungi (Ludley and Robinson 2008). This also accords with the high proportion of Ascomycota in the saprotrophic fungi dataset. Ascomycetes is the most diverse group in the Arctic (Geml et al. 2012; Timling et al. 2014), but is largely unexplored (Dahlberg et al. 2013). Unfortunately, the taxonomic uncertainty in the total fungi dataset was high, so further discussions of our results will be based on the ECM and saprotrophic fungi subsets.

### Deep snow‐induced effects on richness of ECM and saprotrophic fungi

We found a significant decline in ECM fungal richness from control to deep snow treatment, and soil temperature was higher in deep snow plots compared to control (Semenchuk et al. 2013). Our results corroborated a recent winter snow accumulation study, where decrease in ECM fungal richness was observed in dry heath tundra in Alaska (Morgado et al. 2016). These agree with results from previous studies investigating effects of increased summer temperature, in which a clear reduction in ECM richness in moist tundra was also found (Morgado et al. 2014; Geml et al. 2015). Thus, it seems that increasing temperature, regardless of season can have negative effect on ECM richness, but whether the response is short or long term is still unknown.

Soil K, NH_4_‐N, and NO_3_‐N (concentrations are significantly higher in deep snow plots) were related with ECM richness. Although there was no significant difference in soil TON between treatments, this variable represents an important predictor of variation in ECM species composition in the Arctic (Timling et al. 2012). A reduction in ECM fungi with increasing N input has been observed in the Alaskan Arctic (Lilleskov et al. 2002), where several ECM species disappeared completely at high N concentrations and were replaced by other species (Lilleskov et al. 2011). It seems that ECM fungi in the Arctic are sensitive to deep snow‐induced increase in soil N content, a number of ECM OTUs appear unable to resist the resulting changes in soil environmental conditions. We found lower richness of ECM fungi belonging to the genera *Sebacina* and *Hebeloma* under deep snow (Table 3), also correlated with higher N, possibly due to their contact/short‐distance exploration with a smooth mantel, and no or very few emanating hyphae, which relate to greater sensitivity to N deposition (Lilleskov et al. 2011). Conversely, the dominant ECM fungal genera (*Tomentella*,* Cortinarius*, and *Inocybe*) were not affected by deep snow treatment. This accords with the view that more frequent ECM fungi benefit from increased snow depth, increasing their dominance further, whereas rare fungi may suffer (Peers et al. 2012).

We found that saprotrophic fungal richness was significantly higher in the deep snow treatment plots. In support of this, experimentally increased summer temperature has shown to increase decomposition rates (Aerts 2006). In a recent experimental study, summer warming triggered an increase in saprotrophic ascomycetes which were probably favored by increased leaf litter accumulation (Wahren et al. 2005; Walker et al. 2006; Semenova et al. 2014). Further studies are needed to see if differences in litter accumulation between deep snow and control plots drive the enhanced richness in certain saprotrophic groups. We found that richness of saprotrophic genera such as *Ramariopsis* and *Dothidea* was higher in the deep snow treatment, while at the same time many rare OTUs (genera) failed to be recorded in deep snow treatment samples. This may either suggest that rare species become extinct while species richer saprotrophic genera tolerate increased snow conditions. Therefore, under long‐term increased snow conditions, saprotrophic fungal species seem to possess competitive advantages over ECM fungi, which may favor increased dominance in deep snow conditions.

It has been shown that microbial activity and N levels in soil and plant tissue enhance under increased snow conditions (Schimel et al. 2004; Semenchuk et al. 2015). ECM richness was lower under thicker snow cover, despite higher nitrogen availability in the soil and nitrogen accumulation in the plants. Due to increased soil N, host plants may have a lower dependency on ECM symbionts for N mobilization, and consequently allocate less photosynthetic C to fungal partners (Deslippe and Simard 2011). The plants may instead use retained C to build plant biomass. These processes might surpass soil organic matter, and can thereby result in greater richness of the saprotrophic fungi which in turn may enhance decomposition processes and bring about more efficient degradation of recalcitrant substrates and overall ecosystem functioning (Setälä and McLean 2004; van der Wal et al. 2013).

### Temporal variation in richness of ECM and saprotrophic fungi

We observed temporal variation in both communities of saprotrophic and ECM fungi and a peak of fungal richness on 16 August, which is approximately the time when senescence of vascular plants starts for most species in the Arctic environment (Abbandonato et al. unpubl. ms.). This peak in fungal richness is likely due to drawdown of N into the roots. However, air temperature and precipitation also varied with time, and the relative importance of correlated soil variables and weather conditions is difficult to disentangle. In dry Arctic regions, moisture strongly limits primary productivity and increased water availability after precipitation events (and persistent cloud cover that maintains the moist conditions) may trigger brief pulses of resource availability and mycelial growth. In arid desert ecosystems, it has been shown that fungal richness is affected directly by moisture as they are not much resistant to desiccation (Zak et al. 1995); indirectly by moisture‐induced changes in soil chemistry, especially N (Fierer and Schimel 2002), and richness increases due to increased moisture availability (Talley et al. 2002). Soil pH is one of the important factors affecting fungal richness (Tedersoo et al. 2014). Root exudates are known to increase nutrient availability and influence pH, although variation in pH levels is often limited due to buffering capacity at root–soil interface and rhizospheric effect exists only in few mm distance from the root surface (Marschner 2012). This form a distinct local microenvironment and soil pH surrounding the root area is also different than the bulky soil. However, such effects are not pronounced in bulk soil, where pH varies locally. This is most likely the reason why soil pH affected the saprotrophic fungi living in bulk soil, but not symbiotic ECM fungi.

We found inconsistent temporal patterns under deep snow treatment between ECM and saprotrophic (Ascomycetes, Leotiomycetes, and Helotiales) fungi. Overall lower richness of ECM fungi in deep snow samples at all sampling dates can be linked to higher N content in the soil, as discussed earlier. Rewetting of dry soil has been reported to significantly affect C and N mineralization rates (Fierer and Schimel 2002). Increased soil water content due to precipitation (and maintenance of high soil moisture levels by lasting cloud cover) can increase the level of available N in the soil. Consequently, altered nutrient conditions between deep snow and control plots may lead to differential responses of the fungi between snow treatments.

### Community structure in ECM and saprotrophic fungi

The community structure of ECM fungi did not vary over time and was hardly affected by deep snow treatment. It differed among FPC locations being affected by soil NO_3_‐N. This indicates that considerable spatial variation in ECM community structure may be caused by variation in soil water and NO_3_‐N contents, as reported in other studies (Coleman et al. 1989; Lilleskov et al. 2002). High spatial heterogeneity in fungal composition associated with the arctic‐alpine plant *Bistorta vivipara* is reported in several studies from the Arctic (Blaalid et al. 2014; Botnen et al. 2014; Mundra et al. 2015b). Ordination analysis suggested no direct snow treatment effect on ECM communities, but NO_3_‐N was related with the structure. Soil NO_3_‐N was also influenced by increased snow depth, which suggests indirect effect of snow treatment on ECM fungal communities. Another possibility is that the richness of the most frequent ECM genera is unaffected by snow treatment, as indicated by the lack of a treatment effect in the ordination analysis.

In contrast to ECM, saprotrophic communities varied between treatments and fluctuated among sampling dates. The variation in community structure between sampling dates may partly result from fluctuating weather conditions. On 8 and 16 August, some of the most strongly deviating species compositions were observed (see Fig. 3), weather conditions had been particularly warm and wet for the past 7 days. Therefore, it is most likely that many species had better growing conditions in these weeks boosting their biomass temporarily. Furthermore, the communities observed in these dates were richer in species and communities of other sampling dates are subsets of this richer species pool. Thus, weather conditions improving overall growth conditions of fungi also increase the biomass of rare fungal species, increasing detection rate of these species and making them better represented when fungal richness is higher. Such variation in fungal richness can influence the saprotrophic fungal community structure.

The temporal consistency of ECM fungi is most likely due to their extensive mycelium in soils and around short lateral roots of their hosts, which penetrate between epidermal and cortical cells (Peterson et al. 2004). Owing to the lack of mutualistic interactions with higher plants, saprotrophic fungi are expected to be more dependent upon their respective substrates. Therefore, compared with ECM, saprotrophic fungal community are more likely to be influenced by abiotic factors such as soil nutrients or moisture variation (Reverchon et al. 2010).

Both ECM and saprotrophic fungal communities in samples from treatment and control sites responded similarly over time across the growing season, suggesting no temporal phase shifts in both communities, due to increased snow depths in winter or delayed snow melt during start of growing season. This indicates that the deep snow‐induced delay of snow melt has minor or no impact on the temporal variation in fungal communities in our study. Such a phase shift may, however, take place at the start of the growing season or just after snow melt, due to the significant difference in soil water content (Cooper et al. 2011) and chemistry (Semenchuk et al. 2015). This is, however, not addressed in this study, and will be the focus of a future study. Further research is required to explore the fungal responses quantitatively (using, e.g., a biomarker such as ergosterol) as well as using RNA.

Our results show significant and contrasting deep snow treatment effects on richness of different fungal functional groups in an Arctic mesic tundra community (richness of ECM was lower and saprotrophic was higher in deep snow treatment relative to control plots). Such treatment effects are visible at both higher and lower taxonomic level. Indirect deep snow treatment effect was observed for ECM community structure, whereas saprotrophic fungal communities were affected directly by deep snow. We suggest that changing winter precipitation and consequent soil temperature pattern dynamics in the Arctic region are likely to greatly alter soil fungi, which could change the soil functional dynamics by affecting both carbon and nitrogen cycling.

## Conflict of Interest

The authors declare that there are no conflicts of interests.

## Supporting information


**Figure S1.** Boxplots of soil nutrient content with significant treatment effect.
**Figure S2.** Species accumulation curves with their 95% confidence intervals for ECM and saprotrophic fungal dataset.
**Figure S3.** Pattern of temporal variation in total, ectomycorrhizal (ECM), and saprotrophic fungal OTU richness (mean ± SD).
**Figure S4.** Pure and shared effects of snow treatment, sampling date, and environmental variables on communities of different fungal groups, as derived from the variation partitioning analysis.
**Figure S5.** Map showing arrangement of 12 fences in Adventdalen, Svalbard.
**Table S1.** Summary of weather data used for each sampling date.
**Table S2.** Variation in soil variables among the location of fence (deep snow) and paired control (FPC location).
**Table S3.** Percent occurrence of OTUs recovered for different taxonomic groups from ectomycorhizal (ECM) and saprotrophic fungal dataset.
**Table S4.** Fixed effects table for the GLMM fitted to the number of OTUs detected in the samples for total, ectomycorrhizal (ECM), and saprotrophic fungi. GLMM models were run using treatment (deep snow vs. control) and sampling time interaction as fixed‐effect predictor.
**Table S5.** Fixed effects table for the GLMM fitted to the number of operational taxonomic units (OTUs) of saprotrophic fungal taxonomic groups. GLMM models were run using treatment (deep snow vs. control) and sampling time interaction as fixed‐effect predictor.
**Table S6**. Relationships between DCA and GNMDS ordination axes of total, ectomycorrhizal (ECM), and saprotrophic fungal dataset.
**Table S7.** Relationships between GNMDS ordination axis of total, ECM, and saprotrophic fungal OTUs composition and environmental and weather variables.
**Table S8.** Total operational taxonomic unit (OTU) richness of different taxonomic level per treatment (deep snow vs. control) for ectomycorrhizal (ECM) and saprotrophic fungi.
