# Supplementary material for: Ectomycorrhizal and saprotrophic fungi respond differently to long‐term experimentally increased snow depth in the High Arctic
Source: Microbiologyopen. 2016 Jun 2;5(5):856–69. doi: 10.1002/mbo3.375 (PMC5061721; doi:10.1002/mbo3.375)
Supplement: Supplementary file 1 — Figure S1. Boxplots of soil nutrient content with significant treatment effect. Figure S2. Species accumulation curves with their 95% confidence intervals for ECM and saprotrophic fungal dataset. Figure S3. Pattern of temporal variation in total, ectomycorrhizal (ECM), and saprotrophic fungal OTU richness (mean ± SD). Figure S4. Pure and shared effects of snow treatment, sampling date, and environmental variables on communities of different fungal groups, as derived from the variation partitioning analysis. Figure S5. Map showing arrangement of 12 fences in Adventdalen, Svalbard. Table S1. Summary of weather data used for each sampling date. Table S2. Variation in soil variables among the location of fence (deep snow) and paired control (FPC location). Table S3. Percent occurrence of OTUs recovered for different taxonomic groups from ectomycorhizal (ECM) and saprotrophic fungal dataset. Table S4. Fixed effects table for the GLMM fitted to the number of OTUs detected in the samples for total, ectomycorrhizal (ECM), and saprotrophic fungi. GLMM models were run using treatment (deep snow vs. control) and sampling time interaction as fixed‐effect predictor. Table S5. Fixed effects table for the GLMM fitted to the number of operational taxonomic units (OTUs) of saprotrophic fungal taxonomic groups. GLMM models were run using treatment (deep snow vs. control) and sampling time interaction as fixed‐effect predictor. Table S6. Relationships between DCA and GNMDS ordination axes of total, ectomycorrhizal (ECM), and saprotrophic fungal dataset. Table S7. Relationships between GNMDS ordination axis of total, ECM, and saprotrophic fungal OTUs composition and environmental and weather variables. Table S8. Total operational taxonomic unit (OTU) richness of different taxonomic level per treatment (deep snow vs. control) for ectomycorrhizal (ECM) and saprotrophic fungi. [file MBO3-5-856-s001.docx]

## Supporting Information:

Title: Ectomycorrhizal and saptrotrophic fungi respond differently to long-term experimentally increased snow depth in the High Arctic archipelago Svalbard

Authors: Sunil Mundra, Rune Halvorsen, Håvard Kauserud, Mohammad Bahram, Leho Tedersoo, Bo Elberling, Elisabeth J. Cooper, Pernille Bronken Eidesen

Journal : MicrobiologyOpen

## Fig. S1. Boxplots of soil nutrient content with significant treatment effect (deep snow = black; control = grey) are shown here. Significance of treatment was determined using the Student’s t-test at the 5% level, after FDR correction of p-values. Boxes span the lower, median and upper quartiles of the raw data, with the minimum and maximum values are indicated by capped lines. Outliers are indicated by open circles.


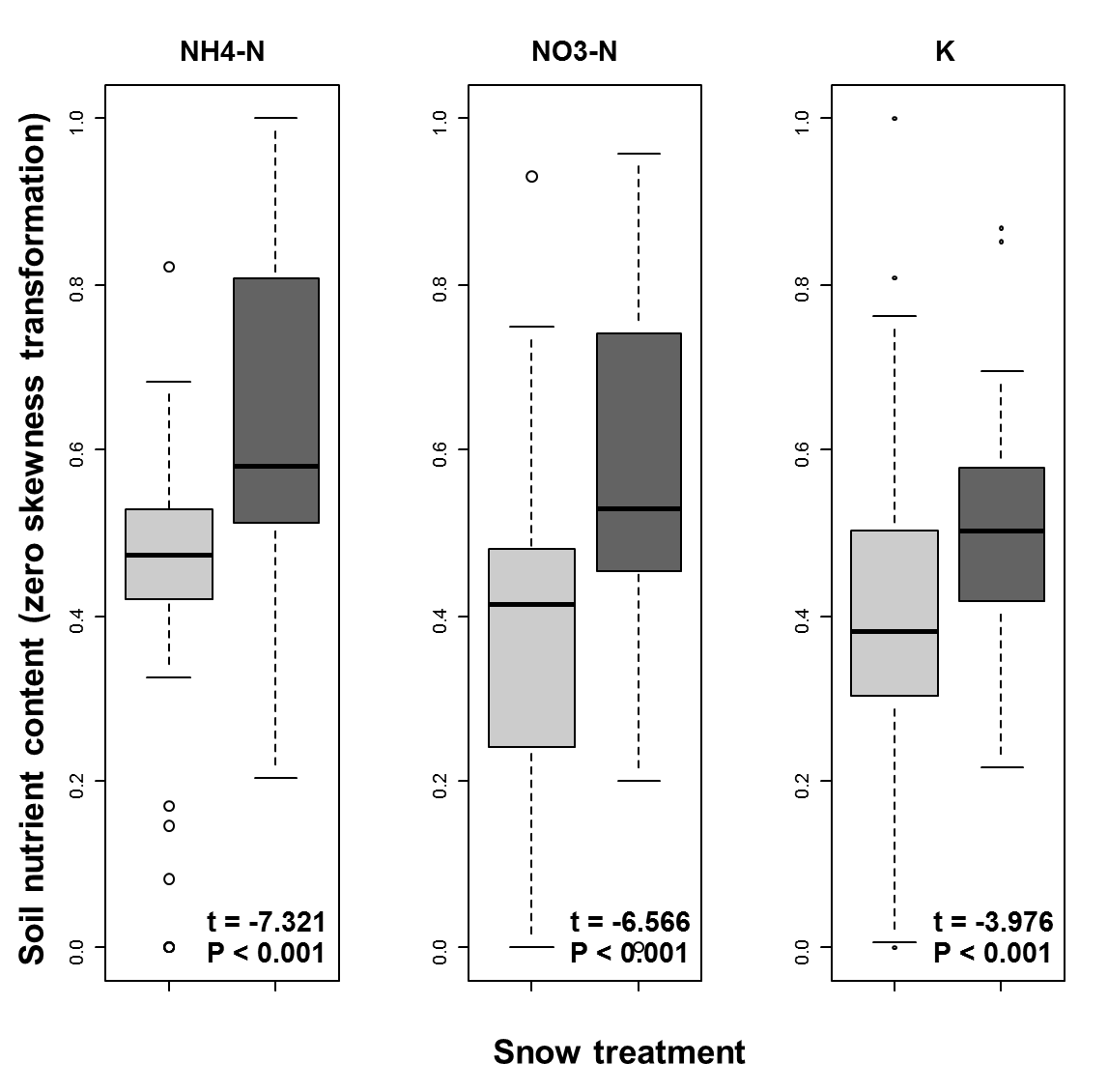


**Fig. S2.** Species accumulation curve and their 95% confidence intervals for ectomycorhizal (ECM; black) and saprotrophic (grey) fungal dataset shown by bar lines (obtained by randomizing 155 samples). Curves for Operational Taxonomic Units (OTUs) against sampling effort were calculated according to Ugland et al. (2003).


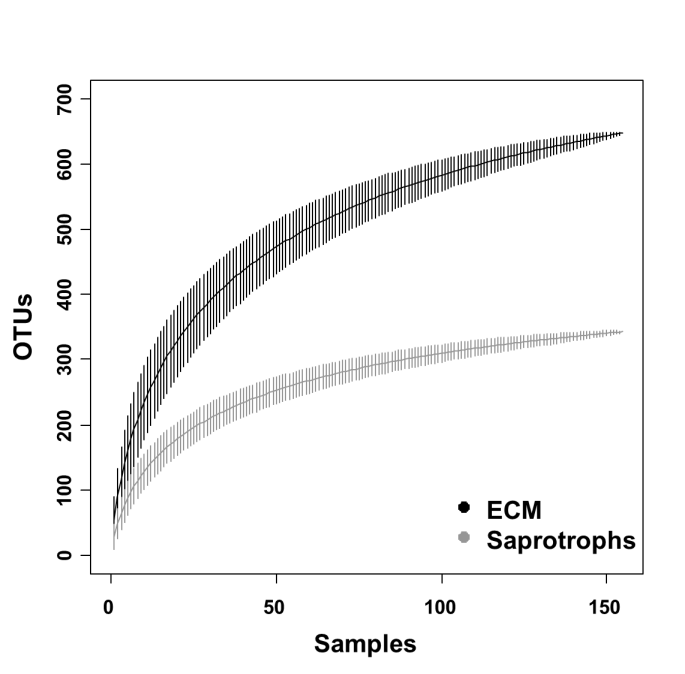


**Fig. S3.** Pattern of temporal variation in fungal (total, ectomycorrhizal (ECM) and saprotrophic) Operational Taxonomic Unit (OTU) richness (mean ± SD). GLMM analyses revealed significant temporal variation in OTU richness of total (z-value=145.4, p<0.001), ECM (z-value=64.8, p<0.001) and saprotrophic fungi (z-value=69.3, p<0.001).


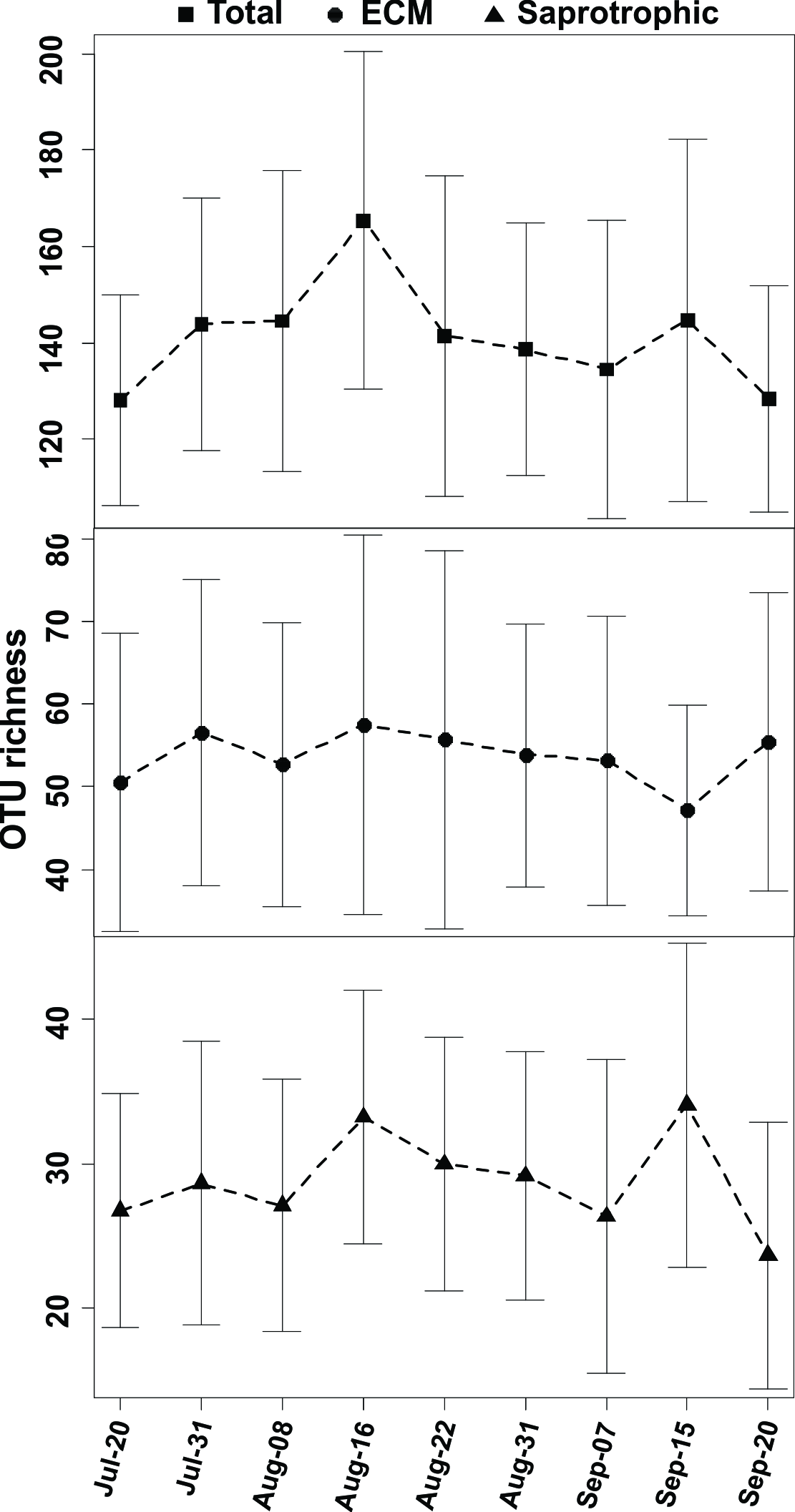


**Fig. S4.** Pure and shared effects of snow treatment (deep snow vs control), sampling date and environmental (both soil and weather) variables on communities of different fungal groups (total, ectomycorrhizal (ECM) and saprotrophic fungi), as derived from the variation partitioning analysis. Numbers indicate the proportion of the explained variation (Adjusted R^2^).


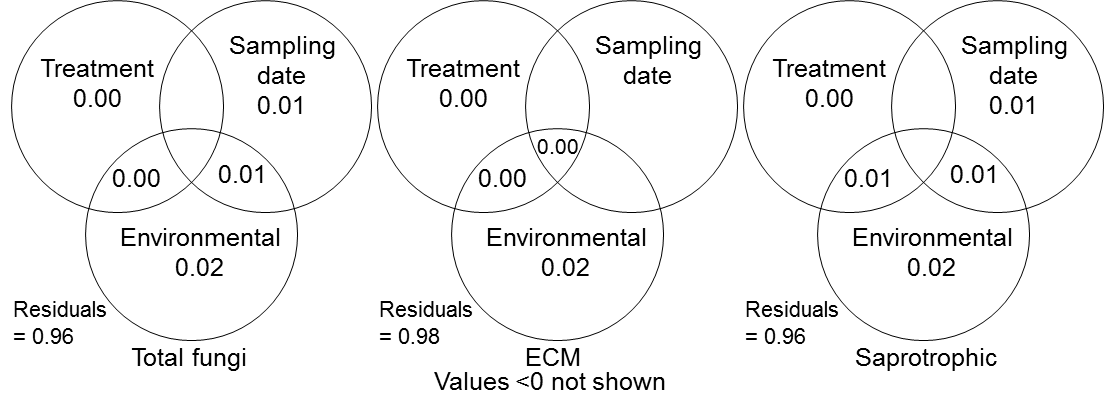


**Fig. S5.** Map showing arrangement of 12 fences (paired control is located at ca. 15-25 m distance from each fence), covering 2.5 km × 1.5 km area, in Adventdalen, Svalbard (six fences in heath vegetation A1-A3, B4-B6; six fences in mesic meadow C7-C9, D10-D12)


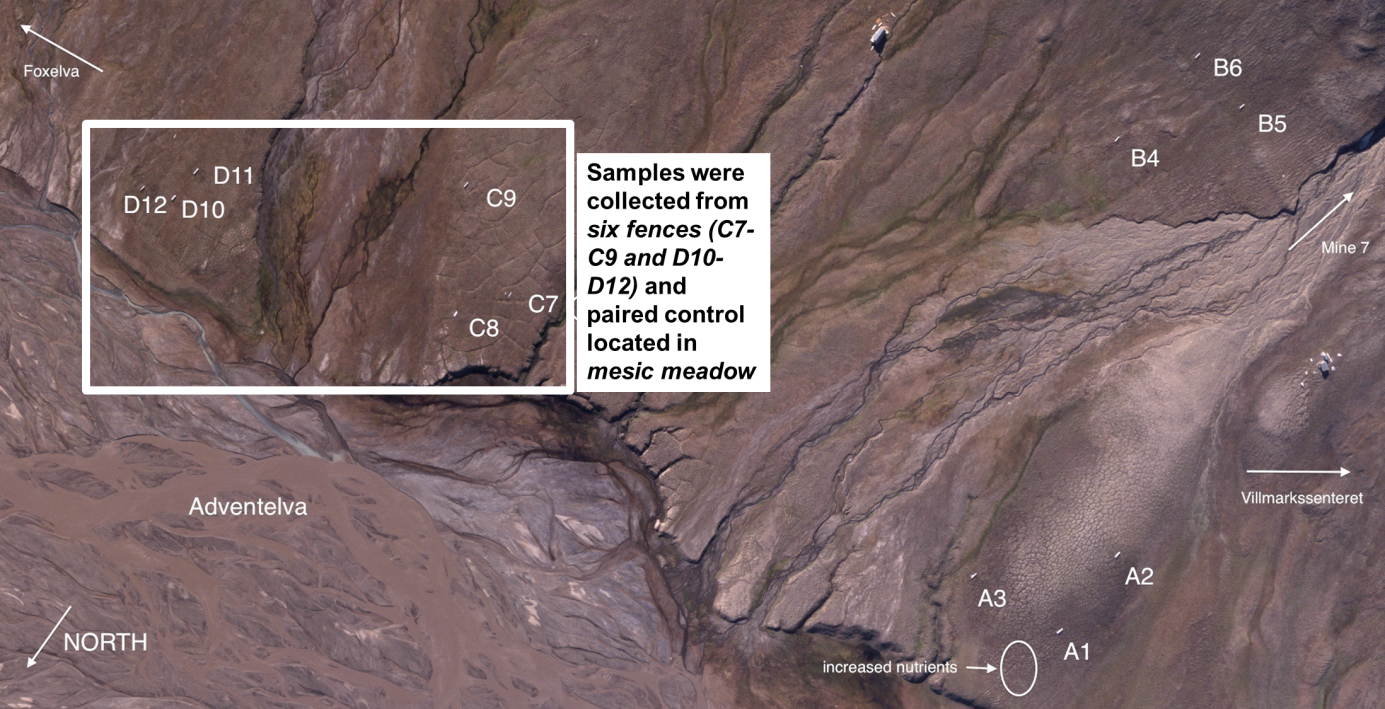


**Table S1.** Summary of weather data used for each sampling date (downloaded from www.eklima.no).

| **Sampling date** | **Minimum air temperature (ºC)** ^1^ | **Precipitation (mm)** ^2^ | **Cloud cover (octas)** ^1^ |
| --- | --- | --- | --- |
| Jul-20 | 5.52 | 0.40 | 5.89 |
| Jul-31 | 4.94 | 2.50 | 6.57 |
| **Aug-08** | **5.07** | **6.30** | **7.14** |
| **Aug-16** | **5.14** | **3.80** | **7.00** |
| Aug-22 | 3.41 | 0.20 | 5.50 |
| Aug-31 | 3.60 | 11.20 | 6.39 |
| Sep-07 | 3.64 | 12.90 | 7.07 |
| Sep-15 | 3.25 | 2.90 | 6.17 |
| Sep-20 | 3.97 | 3.60 | 6.03 |

^1^Average data of the last 7 days, including the sampling date

^2^Cumulative sum of the last 7 days, including the sampling date

**Table S2.** Variation in soil variables among the location of fence (deep snow) and paired control (FPC locations). Significance was determined using an ANOVA test at the 5% level, after FDR correction of p-values. (Soil water: t_5_=9.67, p<0.001 and NO_3_-N content: t_5_=3.87, p=0.002).

| **FPC locations** | **Water content** | **NO_3_-N** |
| --- | --- | --- |
| C7 | 0.46 ± 0.12^ab^ | 0.57 ± 0.22^a^ |
| C8 | 0.55 ± 0.15^a^ | 0.42 ± 0.17^ab^ |
| C9 | 0.56 ± 0.20^a^ | 0.54 ± 0.26^a^ |
| D10 | 0.58 ± 0.19^a^ | 0.53 ± 0.29^ab^ |
| D11 | 0.36 ± 0.17^b^ | 0.42 ± 0.15^ab^ |
| D12 | 0.35 ± 0.14^b^ | 0.36 ± 0.16 ^b^ |

^a-b^Values in a column differ significantly (p<0.05)

**Table S3.** Percent occurrence of Operational Taxonomic Units (OTUs) recovered for different taxonomic groups (phylum, order, and genera) from ectomycorhizal (ECM) and saprotrophic fungal dataset. OTUs obtained from different snow treatment (deep snow vs control) and their overall presences are shown here.

| **Taxonomic group** | **Overall** | **Deep snow** | **Control** |
| --- | --- | --- | --- |
| **ECM fungi** | | | |
| **Basidiomycota** | 96.6% | 96.4% | 96.8% |
| **Ascomycota** | 3.2% | 3.5% | 3.0% |
| **Zygomycota** | 0.2% | 0.2% | 0.2% |
| Agaricales | 51.4% | 51.5% | 51.3% |
| Thelephorales | 31.5% | 31.2% | 31.7% |
| Russulales | 8.9% | 9.1% | 8.8% |
| Cantharellales | 2.9% | 3.1% | 2.8% |
| Sebacinales | 1.9% | 1.6% | 2.2% |
| Hysteriales | 1.2% | 1.3% | 1.2% |
| Pezizales | 1.1% | 1.3% | 1.0% |
| Leotiomycetes_IS | 0.4% | 0.4% | 0.5% |
| Helotiales | 0.4% | 0.5% | 0.3% |
| Endogonales | 0.2% | 0.2% | 0.2% |
| *Tomentella* | 30.9% | 30.9% | 30.8% |
| *Cortinarius* | 30.2% | 28.1% | 31.9% |
| *Inocybe* | 17.2% | 19.4% | 15.4% |
| *Russula* | 5.9% | 5.9% | 6.0% |
| *Hebeloma* | 3.3% | 3.0% | 3.4% |
| *Lactarius* | 3.0% | 3.2% | 2.8% |
| *Clavulina* | 2.9% | 3.1% | 2.8% |
| *Sebacina* | 1.9% | 1.6% | 2.2% |
| *Cenococcum* | 1.2% | 1.3% | 1.2% |
| *Geopora* | 0.7% | 0.9% | 0.6% |
| *Laccaria* | 0.7% | 0.9% | 0.6% |
| *Thelephora* | 0.6% | 0.3% | 0.9% |
| *Meliniomyces* | 0.4% | 0.4% | 0.5% |
| *Meliniomyces* | 0.4% | 0.5% | 0.3% |
| *Pustularia* | 0.3% | 0.4% | 0.2% |
| *Endogone* | 0.2% | 0.2% | 0.2% |
| *Peziza* | 0.1% | 0.0% | 0.2% |
| *Helvella* | 0.0% | 0.0% | 0.0% |
| **Saprotrophic fungi** | | | |
| **Ascomycota** | 47.1% | 46.2% | 48.1% |
| **Basidiomycota** | 39.9% | 41.4% | 38.2% |
| **Zygomycota** | 13.0% | 12.3% | 13.8% |
| Agaricales | 28.0% | 28.8% | 27.1% |
| Mortierellales | 12.9% | 12.2% | 13.7% |
| Chaetothyriales | 11.9% | 11.0% | 13.0% |
| Coniochaetales | 5.7% | 5.1% | 6.4% |
| Pleosporales | 5.3% | 4.9% | 5.8% |
| Helotiales | 4.4% | 4.5% | 4.4% |
| Ascomycota_IS | 4.4% | 4.3% | 4.5% |
| Dothideales | 4.3% | 5.0% | 3.4% |
| Cystofilobasidiales | 3.4% | 3.7% | 3.2% |
| Helotiales | 3.2% | 3.2% | 3.2% |
| Hymenochaetales | 2.4% | 2.5% | 2.2% |
| Auriculariales | 1.8% | 2.1% | 1.5% |
| Eurotiales | 1.7% | 1.6% | 1.9% |
| Hypocreales | 1.6% | 1.5% | 1.7% |
| Sporidiobolales | 1.2% | 1.4% | 1.0% |
| Leotiomycetes_IS | 0.9% | 1.0% | 0.8% |
| Capnodiales | 0.9% | 1.0% | 0.8% |
| Leucosporidiales | 0.7% | 0.5% | 1.0% |
| Dothideomycetes IS | 0.7% | 0.7% | 0.6% |
| Cantharellales | 0.5% | 0.4% | 0.7% |
| Tremellales | 0.5% | 0.6% | 0.5% |
| Thelephorales | 0.5% | 0.6% | 0.3% |
| Filobasidiales | 0.5% | 0.5% | 0.5% |
| Dothideomycetes IS | 0.4% | 0.5% | 0.4% |
| Thelebolales | 0.3% | 0.2% | 0.4% |
| Orbiliales | 0.3% | 0.3% | 0.3% |
| Leotiales | 0.2% | 0.2% | 0.3% |
| Pezizales | 0.2% | 0.3% | 0.1% |
| Sordariales | 0.2% | 0.3% | 0.2% |
| Microthyriales | 0.2% | 0.3% | 0.1% |
| Geoglossales | 0.2% | 0.3% | 0.1% |
| Sebacinales | 0.1% | 0.1% | 0.2% |
| Gomphales | 0.1% | 0.2% | 0.0% |
| Ostropales | 0.1% | 0.1% | 0.1% |
| Agaricostilbales | 0.1% | 0.0% | 0.2% |
| Malasseziales | 0.1% | 0.1% | 0.1% |
| Mucorales | 0.1% | 0.1% | 0.1% |
| Basidiobolales | 0.0% | 0.0% | 0.1% |
| Chaetothyriomycetidae | 0.0% | 0.0% | 0.0% |
| Polyporales | 0.0% | 0.0% | 0.1% |
| *Mortierella* | 12.9% | 12.2% | 13.7% |
| *Cladophialophora* | 8.4% | 7.7% | 9.2% |
| *Mycena* | 5.8% | 5.4% | 6.2% |
| *Lecythophora* | 5.5% | 5.0% | 6.1% |
| *Dothidea* | 4.1% | 4.8% | 3.3% |
| *Ramariopsis* | 4.0% | 4.9% | 3.0% |
| *Mycena* | 3.9% | 4.3% | 3.4% |
| *Mycenella* | 3.4% | 3.7% | 3.0% |
| *Articulospora* | 3.2% | 3.2% | 3.2% |
| *Mrakia* | 3.1% | 3.3% | 2.9% |
| *Galerina* | 2.7% | 2.8% | 2.7% |
| *Chalara* | 2.4% | 2.2% | 2.6% |
| *Sporormiella* | 2.0% | 2.1% | 2.0% |
| *Arrhenia* | 2.0% | 1.8% | 2.3% |
| *Capronia* | 1.8% | 1.7% | 1.9% |
| *Galerina* | 1.7% | 1.2% | 2.3% |
| *Hyphodontia* | 1.6% | 1.5% | 1.7% |
| *Rosasphaeria* | 1.6% | 1.5% | 1.7% |
| *Aspergillus* | 1.5% | 1.4% | 1.7% |
| *Hyaloscypha* | 1.3% | 1.4% | 1.1% |
| *Hyphodontiella* | 1.2% | 1.2% | 1.3% |
| *Hemibeltrania* | 1.2% | 1.0% | 1.4% |
| *Rhodotorula* | 1.2% | 1.4% | 1.0% |
| *Exophiala* | 1.1% | 1.1% | 1.1% |
| *Auricularia* | 1.0% | 1.0% | 1.1% |
| *Rhizoscyphus* | 0.9% | 0.8% | 1.1% |
| *Davidiella* | 0.9% | 0.9% | 0.8% |
| *Phaeosphaeria* | 0.8% | 0.5% | 1.2% |
| *Oliveonia* | 0.8% | 1.1% | 0.4% |
| *Xylodon* | 0.8% | 1.0% | 0.6% |
| *Preussia* | 0.8% | 0.9% | 0.6% |
| *Meliniomyces* | 0.7% | 0.8% | 0.7% |
| *Collybia* | 0.7% | 0.9% | 0.5% |
| *Didymosphaeria* | 0.7% | 0.5% | 1.0% |
| *Tothia* | 0.7% | 0.7% | 0.6% |
| *Leucosporidiella* | 0.6% | 0.4% | 0.9% |
| *Herpotrichia* | 0.6% | 0.4% | 0.8% |
| *Lycoperdon* | 0.6% | 0.5% | 0.7% |
| *Clavaria* | 0.6% | 0.6% | 0.5% |
| *Omphalina* | 0.5% | 0.4% | 0.6% |
| *Cryptosporiopsis* | 0.5% | 0.5% | 0.5% |
| *Cryptococcus* | 0.5% | 0.5% | 0.5% |
| *Phialocephala* | 0.5% | 0.3% | 0.6% |
| *Typhula* | 0.5% | 0.6% | 0.3% |
| *Cryptococcus* | 0.5% | 0.5% | 0.5% |
| *Cadophora* | 0.4% | 0.5% | 0.4% |
| *Sistotrema* | 0.4% | 0.2% | 0.7% |
| *Teberdinia* | 0.4% | 0.4% | 0.4% |
| *Phialophora* | 0.4% | 0.3% | 0.5% |
| *Ochroconis* | 0.4% | 0.6% | 0.1% |
| *Pyrenochaeta* | 0.3% | 0.4% | 0.3% |
| *Mrakiella* | 0.3% | 0.4% | 0.3% |
| *Tricladium* | 0.3% | 0.3% | 0.3% |
| *Entoloma* | 0.3% | 0.3% | 0.4% |
| *Hymenoscyphus* | 0.3% | 0.3% | 0.3% |
| *Thelebolus* | 0.3% | 0.2% | 0.4% |
| *Rhinocladiella* | 0.3% | 0.2% | 0.3% |
| *Crepidotus* | 0.3% | 0.3% | 0.2% |
| *Alatospora* | 0.2% | 0.2% | 0.3% |
| *Tothia* | 0.2% | 0.3% | 0.1% |
| *Emericella* | 0.2% | 0.2% | 0.2% |
| *Cheilymenia* | 0.2% | 0.3% | 0.1% |
| *Gyoerffyella* | 0.2% | 0.3% | 0.1% |
| *Sarcoleotia* | 0.2% | 0.3% | 0.1% |
| *Geomyces* | 0.2% | 0.3% | 0.1% |
| *Chaetomium* | 0.2% | 0.2% | 0.2% |
| *Fontanospora* | 0.2% | 0.1% | 0.3% |
| *Tetracladium* | 0.2% | 0.1% | 0.2% |
| *Dactylellina* | 0.2% | 0.1% | 0.2% |
| *Coniochaeta* | 0.2% | 0.0% | 0.3% |
| *Sebacina* | 0.1% | 0.1% | 0.2% |
| *Ramaria* | 0.1% | 0.2% | 0.0% |
| *Xenochalara* | 0.1% | 0.1% | 0.1% |
| *Cryptodiscus* | 0.1% | 0.1% | 0.1% |
| *Dactylella* | 0.1% | 0.1% | 0.1% |
| *Lepiota* | 0.1% | 0.1% | 0.1% |
| *Lyophyllum* | 0.1% | 0.2% | 0.0% |
| *Ceratobasidium* | 0.1% | 0.1% | 0.1% |
| *Kurtzmanomyces* | 0.1% | 0.0% | 0.2% |
| *Malassezia* | 0.1% | 0.1% | 0.1% |
| *Mastigobasidium* | 0.1% | 0.1% | 0.1% |
| *Mucor* | 0.1% | 0.1% | 0.1% |
| *Dothidea* | 0.1% | 0.1% | 0.1% |
| *Glarea* | 0.1% | 0.1% | 0.0% |
| *Hemimycena* | 0.1% | 0.1% | 0.0% |
| *Phialea* | 0.0% | 0.1% | 0.0% |
| *Dothidea* | 0.0% | 0.1% | 0.0% |
| *Kabatina* | 0.0% | 0.0% | 0.1% |
| *Clavulinopsis* | 0.0% | 0.0% | 0.1% |
| *Psathyrella* | 0.0% | 0.0% | 0.1% |
| *Tulasnella* | 0.0% | 0.1% | 0.0% |
| *Kockovaella* | 0.0% | 0.1% | 0.0% |
| *Basidiobolus* | 0.0% | 0.0% | 0.1% |
| *Cladosporium* | 0.0% | 0.0% | 0.0% |
| *Sydowia* | 0.0% | 0.0% | 0.1% |
| *Gymnostellatospora* | 0.0% | 0.0% | 0.0% |
| *Massariosphaeria* | 0.0% | 0.0% | 0.0% |
| *Sarcinomyces* | 0.0% | 0.0% | 0.0% |
| *Crocicreas* | 0.0% | 0.0% | 0.1% |
| *Clathrosporium* | 0.0% | 0.0% | 0.1% |
| *Lemonniera* | 0.0% | 0.0% | 0.1% |
| *Drechslerella* | 0.0% | 0.0% | 0.0% |
| *Byssonectria* | 0.0% | 0.0% | 0.0% |
| *Fimetariella* | 0.0% | 0.0% | 0.0% |
| *Podospora* | 0.0% | 0.0% | 0.0% |
| *Clitocybe* | 0.0% | 0.0% | 0.0% |
| *Hyphoderma* | 0.0% | 0.0% | 0.1% |

**Table S4.** Fixed effects table for the Generalized Linear Mixed Model (GLMM) fitted to the number of Operational Taxonomic Units (OTUs) detected in the samples for total, ectomycorrhizal (ECM) and saprotrophic fungi. GLMM models were run using treatment (deep snow vs control) and sampling time interaction as fixed-effect predictor while the location of fence (deep snow) and paired control (FPC locations). were included as a random factor in the model. Significance values are shown in bold text.

| **Fixed effects** | **Total fungi** | | | | **ECM fungi** | | | | **Saprotrophic fungi** | | | |
| --- | --- | --- | --- | --- | --- | --- | --- | --- | --- | --- | --- | --- |
|  | **Estimate** | **SE** | **z value** | **Pr(>\|z\|)** | **Estimate** | **SE** | **z value** | **Pr(>\|z\|)** | **Estimate** | **SE** | **z value** | **Pr(>\|z\|)** |
| Intercept | 4.7800 | 0.0401 | 119.2 | **<0.001** | 3.9407 | 0.0688 | 57.3 | **<0.001** | 3.1855 | 0.0689 | 46.3 | **<0.001** |
| Deep snow | 0.1313 | 0.0389 | 3.4 | **0.001** | -0.0535 | 0.0616 | -0.9 | 0.385 | 0.1880 | 0.0854 | 2.2 | **0.028** |
| Jul-31 | 0.0993 | 0.0459 | 2.2 | **0.030** | 0.1398 | 0.0688 | 2.0 | **0.042** | -0.0092 | 0.1053 | -0.1 | 0.931 |
| Aug-08 | 0.2014 | 0.0376 | 5.4 | **<0.001** | 0.1201 | 0.0581 | 2.1 | **0.039** | 0.0514 | 0.0861 | 0.6 | 0.550 |
| Aug-16 | 0.3486 | 0.0427 | 8.2 | **<0.001** | 0.1942 | 0.0678 | 2.9 | **0.004** | 0.2837 | 0.0966 | 2.9 | **0.003** |
| Aug-22 | 0.1534 | 0.0394 | 3.9 | **<0.001** | 0.1562 | 0.0599 | 2.6 | **0.009** | 0.1255 | 0.0883 | 1.4 | 0.155 |
| Aug-31 | 0.0737 | 0.0462 | 1.6 | 0.111 | 0.1258 | 0.0691 | 1.8 | 0.069 | 0.1024 | 0.1018 | 1.0 | 0.314 |
| Sep-07 | 0.0540 | 0.0388 | 1.4 | 0.163 | 0.0674 | 0.0587 | 1.1 | 0.251 | -0.0663 | 0.0884 | -0.8 | 0.453 |
| Sep-15 | 0.2814 | 0.0435 | 6.5 | **<0.001** | 0.0400 | 0.0709 | 0.6 | 0.572 | 0.4055 | 0.0934 | 4.3 | **<0.001** |
| Sep-20 | 0.0008 | 0.0409 | 0.0 | 0.984 | 0.1542 | 0.0600 | 2.6 | **0.010** | -0.2472 | 0.0971 | -2.5 | **0.011** |
| Deep snow:Jul-31 | 0.0370 | 0.0620 | 0.6 | 0.551 | -0.0556 | 0.0985 | -0.6 | 0.572 | 0.1405 | 0.1387 | 1.0 | 0.311 |
| Deep snow:Aug-08 | -0.1440 | 0.0522 | -2.8 | **0.006** | -0.1763 | 0.0848 | -2.1 | **0.038** | -0.0556 | 0.1171 | -0.5 | 0.635 |
| Deep snow:Aug-16 | -0.1767 | 0.0594 | -3.0 | **0.003** | -0.1384 | 0.0982 | -1.4 | 0.159 | -0.1228 | 0.1316 | -0.9 | 0.351 |
| Deep snow:Aug-22 | -0.1114 | 0.0541 | -2.1 | **0.040** | -0.1604 | 0.0861 | -1.9 | 0.063 | -0.0133 | 0.1183 | -0.1 | 0.911 |
| Deep snow:Aug-31 | 0.0156 | 0.0627 | 0.2 | 0.804 | -0.1328 | 0.1002 | -1.3 | 0.185 | -0.0277 | 0.1371 | -0.2 | 0.840 |
| Deep snow:Sep-07 | 0.0177 | 0.0536 | 0.3 | 0.740 | -0.0481 | 0.0853 | -0.6 | 0.573 | 0.1315 | 0.1192 | 1.1 | 0.270 |
| Deep snow:Sep-15 | -0.3173 | 0.0619 | -5.1 | **<0.001** | -0.2383 | 0.1049 | -2.3 | **0.023** | -0.3202 | 0.1308 | -2.4 | **0.014** |
| Deep snow:Sep-20 | 0.0032 | 0.0548 | 0.1 | 0.954 | -0.1234 | 0.0849 | -1.5 | 0.146 | 0.2089 | 0.1257 | 1.7 | 0.096 |

**Table S5.** Fixed effects table for the Generalized Linear Mixed Model (GLMM) fitted to the number of Operational Taxonomic Units (OTUs) of saprotrophic fungal taxonomic groups. GLMM models were run using treatment (deep snow vs control) and sampling time interaction as fixed-effect predictor while location of fence (deep snow) and paired control (FPC locations) were included as a random factor in the model. Significance values are shown in bold text.

| **Fixed effects** | **Ascomycota** | | | | **Helotiales** | | | | **Leotiomycetes** | | | |
| --- | --- | --- | --- | --- | --- | --- | --- | --- | --- | --- | --- | --- |
|  | **Estimate** | **SE** | **z value** | **Pr(>\|z\|)** | **Estimate** | **SE** | **z value** | **Pr(>\|z\|)** | **Estimate** | **SE** | **z value** | **Pr(>\|z\|)** |
| Intercept | 2.1246 | 0.1176 | 18.1 | **<0.001** | -0.1050 | 0.3330 | -0.3 | 0.752 | -1.6120 | 0.7080 | -2.3 | **0.023** |
| Deep snow | 0.2628 | 0.1424 | 1.9 | 0.065 | 0.5980 | 0.4080 | 1.5 | 0.143 | 1.1560 | 0.8010 | 1.4 | 0.149 |
| Jul-31 | 0.2523 | 0.1652 | 1.5 | 0.127 | -0.0770 | 0.5580 | -0.1 | 0.890 | -15.6720 | 418.0490 | 0.0 | 0.970 |
| Aug-08 | 0.4600 | 0.1348 | 3.4 | **0.001** | 1.0200 | 0.3800 | 2.7 | **0.007** | 2.4540 | 0.7310 | 3.4 | **0.001** |
| Aug-16 | 0.9300 | 0.1405 | 6.6 | **<0.001** | 1.8400 | 0.3750 | 4.9 | **<0.001** | 3.5040 | 0.7240 | 4.8 | **<0.001** |
| Aug-22 | 0.4464 | 0.1395 | 3.2 | **0.001** | 0.8470 | 0.3980 | 2.1 | **0.033** | 2.2420 | 0.7430 | 3.0 | **0.003** |
| Aug-31 | 0.3406 | 0.1612 | 2.1 | **0.035** | 0.2600 | 0.5040 | 0.5 | 0.607 | -0.1850 | 1.2240 | -0.2 | 0.880 |
| Sep-07 | 0.2523 | 0.1400 | 1.8 | 0.072 | 0.3930 | 0.4170 | 0.9 | 0.346 | 0.7320 | 0.8360 | 0.9 | 0.382 |
| Sep-15 | 0.9221 | 0.1407 | 6.6 | <0.001 | 1.3600 | 0.3980 | 3.4 | **0.001** | 2.8110 | 0.7410 | 3.8 | **<0.001** |
| Sep-20 | -0.2877 | 0.1666 | -1.7 | 0.084 | 0.0000 | 0.4710 | 0.0 | 1.000 | 0.4050 | 0.9120 | 0.4 | 0.657 |
| Deep snow:Jul-31 | -0.0678 | 0.2198 | -0.3 | 0.758 | 0.0953 | 0.6830 | 0.1 | 0.889 | 14.3310 | 418.0510 | 0.0 | 0.973 |
| Deep snow:Aug-08 | -0.0313 | 0.1791 | -0.2 | 0.861 | -0.0834 | 0.4710 | -0.2 | 0.859 | -0.4400 | 0.8350 | -0.5 | 0.598 |
| Deep snow:Aug-16 | -0.2096 | 0.1884 | -1.1 | 0.266 | -0.7920 | 0.4810 | -1.7 | 0.100 | -1.2080 | 0.8330 | -1.5 | 0.147 |
| Deep snow:Aug-22 | -0.1706 | 0.1864 | -0.9 | 0.360 | -0.5070 | 0.5080 | -1.0 | 0.318 | -1.3860 | 0.8730 | -1.6 | 0.112 |
| Deep snow:Aug-31 | -0.0482 | 0.2138 | -0.2 | 0.822 | -0.3470 | 0.6490 | -0.5 | 0.593 | 0.2300 | 1.3750 | 0.2 | 0.867 |
| Deep snow:Sep-07 | 0.0290 | 0.1872 | 0.2 | 0.877 | 0.0308 | 0.5190 | 0.1 | 0.953 | 0.3190 | 0.9470 | 0.3 | 0.736 |
| Deep snow:Sep-15 | -0.7250 | 0.2016 | -3.6 | **<0.001** | -1.4500 | 0.5700 | -2.5 | **0.011** | -2.2060 | 0.9140 | -2.4 | **0.016** |
| Deep snow:Sep-20 | 0.3124 | 0.2102 | 1.5 | 0.137 | -0.2510 | 0.5910 | -0.4 | 0.671 | -0.9650 | 1.1070 | -0.9 | 0.383 |

**Table S6**. Relationships between DCA and GNMDS ordination axes of total, ectomycorrhizal (ECM) and saprotrophic fungal dataset, assessed by Kendall’s rank correlation coefficients (|τ|). The null hypothesis that there is no relationship between axes in each pair is tested against the two-tailed alternative hypothesis. Correlation coefficients between axes of ≥0.40 (corresponding to α = 0.05 in tests of the hypothesis that |τ| = 0) are indicated by bold font.

| **Multiple parallel ordinations** | | | | |
| --- | --- | --- | --- | --- |
|  | **DCA1** | | **DCA2** | |
|  | \|τ\| | p-value | \|τ\| | p-value |
| **Total fungi** | | | | |
| **GNMDS1** | **-0.799** | **<0.0001** | 0.562 | -0.0314 |
| **GNMDS2** | -0.031 | 0.568 | **-0.704** | **<0.0001** |
| **ECM fungi** | | | | |
| **GNMDS1** | **-0.598** | **<0.0001** | -0.173 | 0.0014 |
| **GNMDS2** | 0.11 | 0.0431 | **-0.695** | **<0.0001** |
| **Saprotrophic fungi** | | | | |
| **GNMDS1** | **-0.527** | **<0.0001** | 0.127 | 0.0198 |
| **GNMDS2** | -0.027 | 0.616 | **-0.683** | **<0.0001** |

**Table S7.** Relationships between GNMDS ordination axis of total, ectomycorrhizal (ECM) and saprotrophic fungal OTUs composition and environmental and weather variables, assessed by Kendall's rank correlation coefficients (|τ|). P-values refer to tests of the hypotheses that τ = 0. Correlation coefficients τ ≥0.20, corresponding to α = 0.05 (in tests of the hypothesis that |τ| = 0) are indicated by bold font. The "variance" columns give the bivariate coefficients of determination r^2^ for the regression of the variable in question of GNMDS axes 1 and 2, as obtained by the *envfit* procedure in *vegan*, and the associated p values obtained by a Monte Carlo randomisation test. Significance of factor (location of fence (deep snow) and paired control (FPC location), treatment, sampling date, and depth) and vector variables were determined after Bonferroni correction of p-values.

| **Parameter** | **Total fungi** | | | | | | **ECM fungi** | | | | | | **Saprotrophic fungi** | | | | | |
| --- | --- | --- | --- | --- | --- | --- | --- | --- | --- | --- | --- | --- | --- | --- | --- | --- | --- | --- |
|  | Kendall (\|τ\|) correlation coefficient | | | |  |  | Kendall (\|τ\|) correlation coefficient | | | |  |  | Kendall (\|τ\|) correlation coefficient | | | |  |  |
|  | gnmds1 | | gnmds2 | | Variance | | gnmds1 | | gnmds2 | | Variance | | gnmds1 | | gnmds2 | | Variance | |
|  | \|τ\| | p | \|τ\| | p | r2 | Pr(>r) | \|τ\| | p | \|τ\| | p | r2 | Pr(>r) | \|τ\| | p | \|τ\| | p | r2 | Pr(>r) |
| **Soil pH** | 0.006 | 0.9193 | 0.041 | 0.4724 | 0.003 | 0.788 | 0.010 | 0.8602 | 0.013 | 0.8188 | 0.000 | 0.983 | -0.028 | 0.6156 | -0.017 | 0.7589 | 0.001 | 0.927 |
| **Water content** | -0.154 | 0.0046 | -0.087 | 0.1096 | 0.056 | 0.010 | **0.243** | **0.0000** | -0.087 | 0.1096 | **0.130** | **0.001** | 0.107 | 0.0492 | 0.013 | 0.8062 | 0.018 | 0.253 |
| **Soil NO3** | -0.184 | 0.0007 | -0.054 | 0.3160 | 0.056 | 0.015 | 0.161 | 0.0031 | 0.125 | 0.0213 | **0.079** | **0.006** | -0.105 | 0.0544 | 0.126 | 0.0203 | 0.034 | 0.067 |
| **Soil NH4** | -0.133 | 0.0144 | 0.030 | 0.5828 | 0.041 | 0.047 | 0.084 | 0.1207 | 0.023 | 0.6682 | 0.046 | 0.037 | -0.027 | 0.6160 | 0.094 | 0.0826 | 0.013 | 0.383 |
| **Soil TON** | 0.086 | 0.1131 | 0.105 | 0.0539 | 0.045 | 0.026 | -0.093 | 0.0853 | -0.005 | 0.9211 | 0.022 | 0.191 | -0.020 | 0.7135 | -0.096 | 0.0778 | 0.022 | 0.198 |
| **Soil TOC** | -0.026 | 0.6249 | 0.059 | 0.2774 | 0.008 | 0.517 | -0.006 | 0.9089 | 0.044 | 0.4175 | 0.004 | 0.763 | 0.032 | 0.5590 | 0.024 | 0.6641 | 0.004 | 0.719 |
| **Soil K** | -0.177 | 0.0011 | 0.095 | 0.0804 | 0.119 | **0.001** | 0.093 | 0.0853 | 0.048 | 0.3795 | 0.050 | 0.014 | 0.048 | 0.3766 | 0.105 | 0.0531 | 0.051 | 0.024 |
| **Temp at 2cm** | 0.012 | 0.8320 | -0.027 | 0.6236 | 0.001 | 0.929 | -0.052 | 0.3379 | 0.032 | 0.5594 | 0.010 | 0.447 | -0.066 | 0.2324 | -0.022 | 0.6867 | 0.007 | 0.602 |
| **Max air temp** | -0.064 | 0.2648 | 0.039 | 0.4955 | 0.020 | 0.205 | 0.003 | 0.9540 | -0.035 | 0.5358 | 0.001 | 0.915 | 0.060 | 0.2935 | 0.007 | 0.9035 | 0.013 | 0.343 |
| **Min air temp** | 0.046 | 0.4236 | -0.177 | 0.0020 | 0.053 | 0.025 | 0.004 | 0.9391 | -0.037 | 0.5154 | 0.000 | 0.982 | -0.075 | 0.1896 | -0.050 | 0.3787 | 0.026 | 0.120 |
| **Cloud cover** | 0.093 | 0.1018 | **-0.283** | 0.0000 | 0.181 | **0.001** | 0.014 | 0.8017 | -0.029 | 0.6078 | 0.002 | 0.829 | -0.146 | 0.0110 | -0.092 | 0.1092 | **0.066** | **0.007** |
| **Precipitation** | 0.053 | 0.3485 | -0.167 | 0.0034 | 0.007 | 0.611 | 0.018 | 0.7539 | -0.047 | 0.4129 | 0.006 | 0.666 | -0.030 | 0.6022 | -0.068 | 0.2332 | 0.015 | 0.317 |
| **FPC locations** |  |  |  |  | 0.126 | **0.001** |  |  |  |  | 0.333 | **0.001** |  |  |  |  | **0.085** | **0.004** |
| **Sampling date** |  |  |  |  | 0.205 | **0.001** |  |  |  |  | 0.022 | 0.989 |  |  |  |  | **0.197** | **0.001** |
| **Snow treatment** |  |  |  |  | 0.014 | 0.118 |  |  |  |  | 0.026 | 0.023 |  |  |  |  | **0.041** | **0.003** |
| **Sampling depth** |  |  |  |  | 0.022 | 0.039 |  |  |  |  | 0.005 | 0.500 |  |  |  |  | 0.006 | 0.393 |

**Table S8**. Total Operational Taxonomic Unit (OTU) richness of different taxonomic level per treatment (deep snow vs control), for ectomycorrhizal (ECM) and saprotrophic fungi.

| **Taxonomic group** | **Deep snow** | | **Control** |
| --- | --- | --- | --- |
| **ECM fungi** | | | |
| Basidiomycota | 3656 | | 4366 |
| Ascomycota | 131 | | 136 |
| Agaricomycetes | 3656 | | 4366 |
| Dothideomycetes | 48 | | 54 |
| Pezizomycetes | 50 | | 45 |
| Leotiomycetes | 33 | | 37 |
| Agaricales | 1953 | | 2313 |
| Thelephorales | 1184 | | 1428 |
| Russulales | 344 | | 397 |
| Cantharellales | 116 | | 127 |
| Sebacinales | 59 | | 101 |
| Hysteriales | 48 | | 54 |
| Pezizales | 50 | | 45 |
| Thelephoraceae | 1184 | | 1428 |
| Cortinariaceae | 1067 | | 1437 |
| Inocybaceae | 737 | | 693 |
| Russulaceae | 344 | | 397 |
| Strophariaceae | 115 | | 155 |
| Clavulinaceae | 116 | | 127 |
| Sebacinaceae | 59 | | 101 |
| Gloniaceae | 48 | | 54 |
| Pyronemataceae | 49 | | 37 |
| Tomentella | 1173 | | 1389 |
| Cortinarius | 1067 | | 1437 |
| Inocybe | 737 | | 693 |
| Russula | 222 | | 271 |
| Hebeloma | 115 | | 155 |
| Lactarius | 122 | | 126 |
| Clavulina | 116 | | 127 |
| Sebacina | 59 | | 101 |
| Cenococcum | 48 | | 54 |
| Geopora | 35 | | 27 |
| Laccaria | 34 | | 28 |
| Thelephora | 11 | | 39 |
| **Saprotrophic fungi** | | | |
| Ascomycota | | 1093 | 961 |
| Basidiomycota | | 980 | 763 |
| Zygomycota | | 292 | 276 |
| Agaricomycetes | | 821 | 638 |
| Eurotiomycetes | | 299 | 296 |
| Zygomycota class Incertae sedis | | 292 | 276 |
| Dothideomycetes | | 291 | 221 |
| Sordariomycetes | | 238 | 227 |
| Leotiomycetes | | 146 | 118 |
| Tremellomycetes | | 112 | 81 |
| Agaricales | | 680 | 541 |
| Mortierellales | | 288 | 274 |
| Chaetothyriales | | 260 | 259 |
| Coniochaetales | | 120 | 127 |
| Pleosporales | | 115 | 116 |
| Helotiales | | 182 | 152 |
| Ascomycota order Incertae sedis | | 102 | 90 |
| Dothideales | | 118 | 68 |
| Cystofilobasidiales | | 87 | 63 |
| Hymenochaetales | | 59 | 44 |
| Auriculariales | | 50 | 29 |
| Eurotiales | | 38 | 37 |
| Hypocreales | | 35 | 33 |
| Sporidiobolales | | 32 | 19 |
| Mortierellaceae | | 288 | 274 |
| Herpotrichiellaceae | | 260 | 259 |
| Tricholomataceae | | 263 | 196 |
| Clavariaceae | | 159 | 96 |
| Mycenaceae | | 131 | 123 |
| Coniochaetaceae | | 120 | 127 |
| Strophariaceae | | 94 | 99 |
| Ascomycota family Incertae sedis | | 102 | 90 |
| Dothideaceae | | 115 | 65 |
| Cystofilobasidiaceae | | 87 | 63 |
| Helotiaceae | | 110 | 97 |
| Sporormiaceae | | 71 | 51 |
| Schizoporaceae | | 59 | 44 |
| Trichocomaceae | | 38 | 37 |
| Niessliaceae | | 35 | 33 |
| Hyaloscyphaceae | | 34 | 21 |
| Sporidiobolales family Incertae sedis | | 32 | 19 |
| Auriculariales family Incertae sedis | | 24 | 21 |
| Pleosporales family Incertae sedis | | 20 | 22 |
| Leotiomycetes family Incertae sedis | | 24 | 16 |
| Mortierella | | 288 | 274 |
| Cladophialophora | | 183 | 184 |
| Mycena | | 229 | 191 |
| Lecythophora | | 119 | 121 |
| Dothidea | | 113 | 65 |
| Ramariopsis | | 115 | 59 |
| Mycenella | | 87 | 60 |
| Articulospora | | 76 | 64 |
| Mrakia | | 77 | 58 |
| Galerina | | 66 | 53 |
| Chalara | | 53 | 51 |
| Sporormiella | | 50 | 39 |
| Arrhenia | | 42 | 46 |
| Capronia | | 40 | 38 |
| Hyphodontia | | 36 | 33 |
| Rosasphaeria | | 35 | 33 |
| Aspergillus | | 33 | 33 |
| Hyaloscypha | | 34 | 21 |
| Hyphodontiella | | 29 | 25 |
| Hemibeltrania | | 23 | 28 |
| Rhodotorula | | 32 | 19 |
| Exophiala | | 25 | 21 |
